# Complement C3a treatment accelerates recovery after stroke via modulation of astrocyte reactivity and cortical connectivity

**DOI:** 10.1172/JCI162253

**Published:** 2023-05-15

**Authors:** Anna Stokowska, Markus Aswendt, Daniel Zucha, Stephanie Lohmann, Frederique Wieters, Javier Morán Suarez, Alison L. Atkins, YiXian Li, Maria Miteva, Julia Lewin, Dirk Wiedermann, Michael Diedenhofen, Åsa Torinsson Naluai, Pavel Abaffy, Lukas Valihrach, Mikael Kubista, Mathias Hoehn, Milos Pekny, Marcela Pekna

**Affiliations:** 1 Laboratory of Regenerative Neuroimmunology, Center for Brain Repair, Department of Clinical Neuroscience, Institute of Neuroscience and Physiology, Sahlgrenska Academy at the University of Gothenburg, Gothenburg, Sweden.; 2 Department of Neurology, Faculty of Medicine, University of Cologne, and University Hospital Cologne, Cologne, Germany.; 3 Laboratory of Gene Expression, Institute of Biotechnology, Czech Academy of Sciences, Prague, Czech Republic.; 4 Department of Informatics and Chemistry, Faculty of Chemical Technology, University of Chemistry and Technology, Prague, Czech Republic.; 5 Multimodal Imaging Group, Max Planck Institute for Metabolism Research, Cologne, Germany.; 6 Department of Laboratory Medicine, Institute of Biomedicine, Sahlgrenska Academy at the University of Gothenburg, Gothenburg, Sweden.; 7 Cognitive Neuroscience, Institute of Neuroscience and Medicine (INM-3), Research Center Juelich, Juelich, Germany.; 8 Laboratory of Astrocyte Biology and CNS Regeneration, Center for Brain Repair, Department of Clinical Neuroscience, Institute of Neuroscience and Physiology, Sahlgrenska Academy at the University of Gothenburg, Gothenburg, Sweden.

**Keywords:** Neuroscience, Complement, Stroke

## Abstract

Despite advances in acute care, ischemic stroke remains a major cause of long-term disability. Approaches targeting both neuronal and glial responses are needed to enhance recovery and improve long-term outcome. The complement C3a receptor (C3aR) is a regulator of inflammation with roles in neurodevelopment, neural plasticity, and neurodegeneration. Using mice lacking C3aR (*C3aR^–/–^*) and mice overexpressing C3a in the brain, we uncovered 2 opposing effects of C3aR signaling on functional recovery after ischemic stroke: inhibition in the acute phase and facilitation in the later phase. Peri-infarct astrocyte reactivity was increased and density of microglia reduced in *C3aR^–/–^* mice; C3a overexpression led to the opposite effects. Pharmacological treatment of wild-type mice with intranasal C3a starting 7 days after stroke accelerated recovery of motor function and attenuated astrocyte reactivity without enhancing microgliosis. C3a treatment stimulated global white matter reorganization, increased peri-infarct structural connectivity, and upregulated *Igf1* and *Thbs4* in the peri-infarct cortex. Thus, C3a treatment from day 7 after stroke exerts positive effects on astrocytes and neuronal connectivity while avoiding the deleterious consequences of C3aR signaling during the acute phase. Intranasal administration of C3aR agonists within a convenient time window holds translational promise to improve outcome after ischemic stroke.

## Graphical abstract

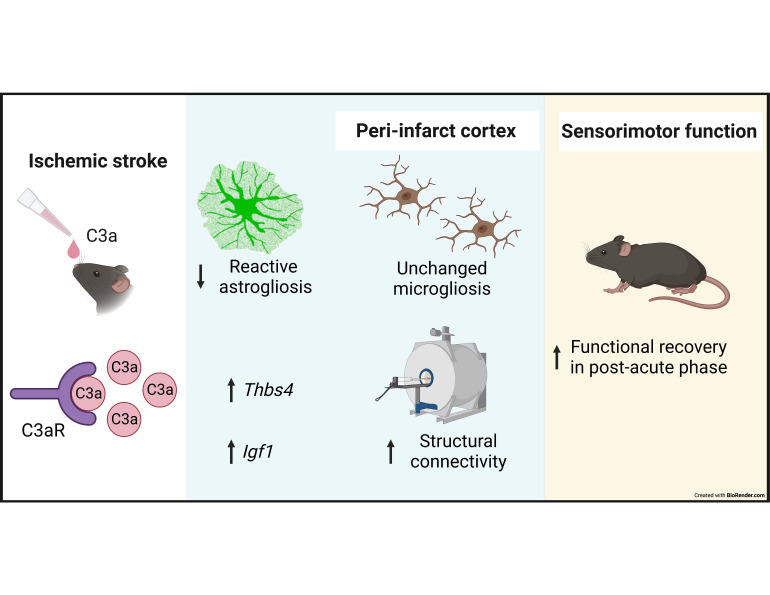

## Introduction

Ischemic stroke affects around 7.6 million people each year with global prevalence of over 77 million (1). Despite the major advances in acute stroke treatment and care, more than 50% of stroke survivors experience long-term sequelae, including loss of voluntary movement in the arm and leg, speech disturbances, depression and anxiety, cognitive deficits, epilepsy, and gait instability and requiring institutional care or long-term assistance (2). Understanding the cellular and molecular mechanisms that contribute to functional recovery could lead to novel treatment approaches to promote recovery, improve long-term outcomes, and reduce the socioeconomic burden of stroke (3, 4).

Reactive gliosis — a dynamic, highly orchestrated response of astrocytes and microglia — limits tissue loss and restores tissue homeostasis after ischemic injury in the central nervous system (CNS). Reactive astrocytes also impact ischemia-induced plasticity and functional recovery (5–8). Transcriptomic changes in astrocytes are context and region dependent and drastically change over time (9, 10). Within hours after the onset of ischemia, astrocytes upregulate their expression of glial fibrillary acidic protein (GFAP), a hallmark of astrocyte reactivity (4, 11). Within days, astrocytes in the peri-infarct region undergo hypertrophy of their cellular processes, forming a glial scar around the damaged area to prevent infiltrating leukocytes from spreading into surrounding healthy parenchyma (4, 12–14). Microglia in the periphery of the ischemic lesion become activated as early as 30 minutes after ischemia onset (15), and the density of activated microglia/macrophages increases for several weeks thereafter (16). The expression profile of microglia/macrophages in the peri-infarct region suggests a dynamic transition from a neuroprotective phenotype in the early stage after stroke to a detrimental phenotype in the later stage (16, 17). Timely modulation of reactive gliosis thus appears to be a rational approach to promote recovery and improve outcome; however, the pharmacological tools to achieve that are lacking.

The complement C3a receptor (C3aR) is a G protein–coupled receptor broadly expressed in the CNS, including astrocytes, microglia, and neural stem cells (18). C3aR signaling has a role in CNS development (19–21) and exerts a range of effects on neural cells. In vitro, C3a, a peptide generated by proteolytic activation of the third complement component (C3), upregulates the expression of nerve growth factor in microglia and astrocytes (22, 23), increases the survival of astrocytes, and reduces their expression of GFAP after ischemic stress (24). C3a also regulates the proliferation, migration, and differentiation of neural progenitor cells in vitro (25), and C3aR signaling stimulates neurogenesis in unchallenged adult mice (26). Signaling through C3aR stimulates neural plasticity in peri-infarct cortex after ischemic stroke (27, 28). However, C3aR can also contribute to neuropathology. C3aR has been implicated in Alzheimer’s disease–type neurodegeneration (29, 30), virus-induced synapse loss and memory impairment (31), and blood-brain barrier dysfunction associated with aging (32).

In this study, we investigated the role of the C3a/C3aR axis in regulating glial responses in the peri-infarct cortex of mice subjected to ischemic stroke. We also assessed functional recovery and used in vivo T2-weighted magnetic resonance imaging and diffusion tensor imaging to determine the effects of intranasal C3a administration on global and peri-infarct connectivity.

## Results

### Increased astrocyte reactivity and reduced microglia density in peri-infarct cortex and improved recovery in the acute phase after stroke in C3aR^–/–^ mice.

**Figure 1:**
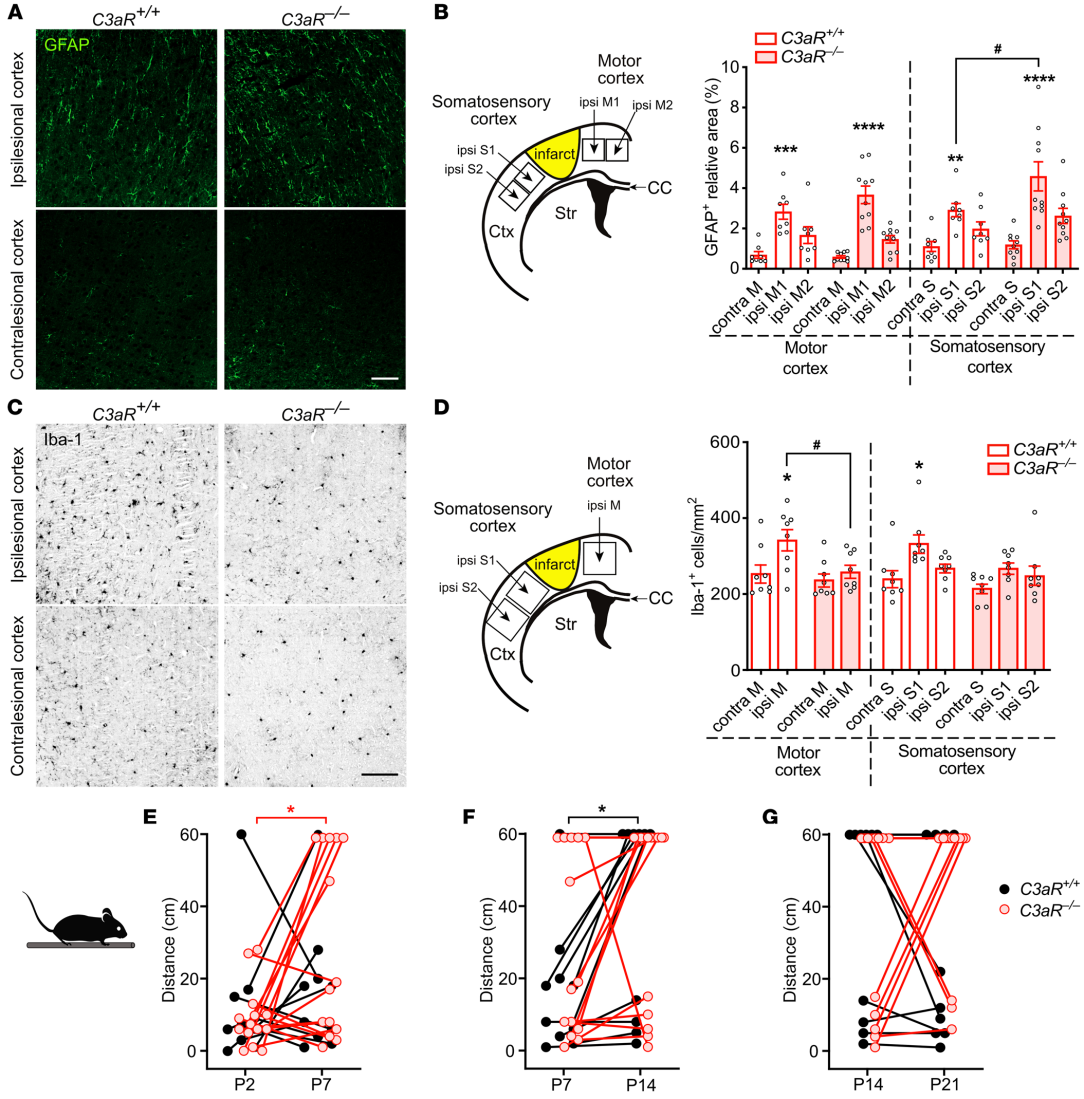
*C3aR^–/–^* mice have increased astrocyte reactivity and reduced density of microglia in peri-infarct cortex. (**A**) Representative images of ipsilesional and contralesional cortex of *C3aR^+/+^* and *C3aR^–/–^* mice in which astrocytes are visualized with antibodies against GFAP on P21. Scale bar: 100 μm. (**B**) Schematic of cortical regions chosen for analysis (left) and relative GFAP-positive area in proximal peri-infarct and contralesional cortex (right). *C3aR^+/+^*, *n* = 8; *C3aR^–/–^*, *n* = 10. Ctx, cortex; CC, corpus callosum; Str, striatum; contra, contralesional; ipsi, ipsilesional; M, motor cortex; S, somatosensory cortex. (**C**) Representative images of ipsilesional and contralesional cortex of *C3aR^+/+^* and *C3aR^–/–^* mice stained with antibodies against Iba-1 on P21. Scale bar: 100 μm. (**D**) Schematic of cortical regions chosen for analysis (left) and density of Iba-1–positive cells in the proximal peri-infarct and contralesional cortex (right). *C3aR^+/+^*, *n* = 8; *C3aR^–/–^*, *n* = 8. (**E**–**G**) Recovery of motor function of *C3aR^+/+^* (*n* = 10) and *C3aR^–/–^* (*n* = 14) mice as assessed by changes in distance walked in the beam test between P2 and P7 (**E**), P7 and P14 (**F**), and P14 and P21 (**G**). Bar plots represent mean ± SEM. Two-way ANOVA with Šidák’s planned comparisons (**B** and **D**): *P < 0.05, **P < 0.01, ***P < 0.001, ****P < 0.0001 for ipsilesional vs. contralesional comparisons; ^#^*P* < 0.05 for between-genotype comparisons. Two-way ANOVA with repeated measures and Šidák’s planned comparisons (**E** and **F**): *P < 0.05.

To assess the role of C3aR signaling in regulating astrocyte reactivity after stroke, we subjected *C3aR^–/–^* and WT *C3aR^+/+^* mice to focal cerebral ischemia in the left cortex at the border between primary motor and primary somatosensory cortical areas corresponding to the forelimb. Reactive astrocytes were visualized by immunostaining with antibodies against the astrocyte marker GFAP (33) (Figure 1A). Twenty-one days after induction of ischemic injury, the GFAP-positive area (quantified by high-content image analysis) was larger in peri-infarct motor and somatosensory cortex than in the corresponding regions of the contralesional hemisphere in both *C3aR^+/+^* and *C3aR^–/–^* mice (Figure 1B). GFAP expression in peri-infarct somatosensory cortex was higher in the *C3aR^–/–^* mice (*P* < 0.05). GFAP expression in the motor cortex did not differ between groups (Figure 1B).

Next, we assessed the density of cells positive for ionized calcium-binding adapter molecule 1 (Iba1), a marker of resident microglia and blood-derived macrophages, in peri-infarct cortex (Figure 1, C and D). In *C3aR^+/+^* mice, but not *C3aR^–/–^* mice, the density of Iba-1–positive cells was higher in peri-infarct motor and somatosensory cortex than in the contralesional hemisphere (*P* < 0.05). The density of Iba-1–positive cells in peri-infarct motor cortex was lower in *C3aR^–/–^* than in *C3aR^+/+^* mice (*P* < 0.05; Figure 1D).

These results show that signaling through the C3aR downregulates GFAP expression in peri-infarct astrocytes. As monocyte-derived macrophages do not persist in the peri-infarct region beyond post-stroke day (P) 8 (34), our findings suggest that C3aR signaling plays a role in microglia migration and/or proliferation in perilesional parenchyma.

Motor function improved between P2 and P7 in *C3aR^–/–^* mice (*P* < 0.05; Figure 1E) and between P7 and P14 in *C3aR^+/+^* mice (*P* < 0.05; Figure 1F) but did not improve in either group between P14 and P21 (Figure 1G). Since infarct volume was not affected by the genetic absence of C3aR (*C3aR^–/–^*, 1.34 ± 0.34 mm^3^, vs. *C3aR^+/+^*, 1.07 ± 0.23 mm^3^, *P* = 0.124), these findings suggest a role for C3aR signaling in the dynamics of functional recovery.

### Reduced astrocyte reactivity and increased microglia density in peri-infarct cortex and improved recovery in the postacute phase after stroke in mice overexpressing C3a in the brain.

**Figure 2:**
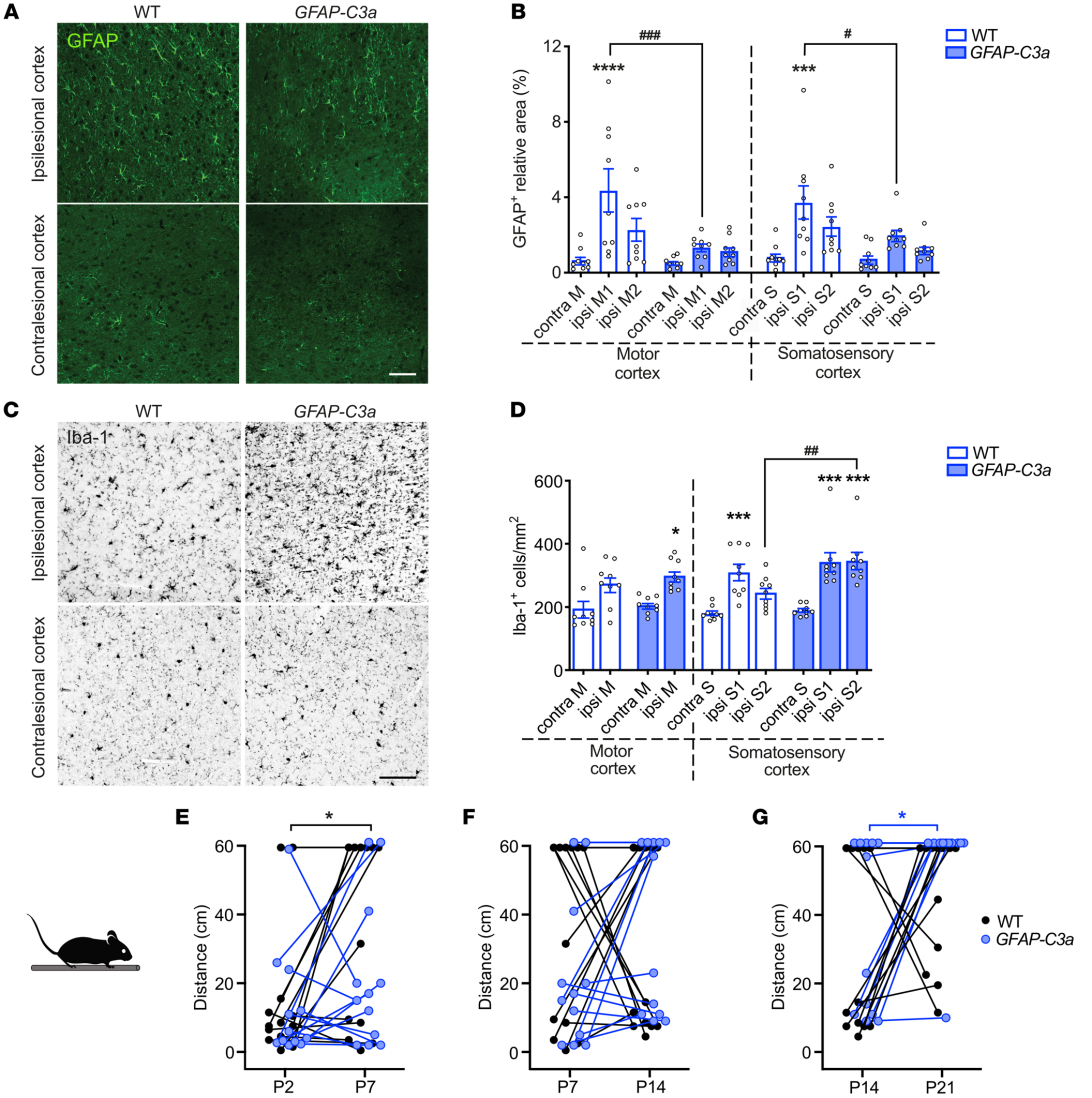
Overexpression of C3a reduces astrocyte reactivity but increases microglia density in peri-infarct cortex. (**A**) Representative images of ipsilesional and contralesional cortex of WT and *GFAP*-*C3a* mice in which astrocytes are visualized with antibodies against GFAP on P21. Scale bar: 100 μm. (**B**) Relative GFAP-positive area in proximal peri-infarct and contralesional cortex. WT, *n* = 9; *GFAP-C3a*, *n* = 9. Contra, contralesional; ipsi, ipsilesional; M, motor cortex; S, somatosensory cortex. Regions for analysis were chosen as shown in Figure 1B. (**C**) Representative images of ipsilesional and contralesional cortex of WT and *GFAP*-*C3a* mice stained with antibodies against Iba-1 on P21. Scale bar: 100 μm. (**D**) Density of Iba-1–positive cells in proximal peri-infarct and contralesional cortex. WT, *n* = 9; *GFAP-C3a*, *n* = 9. Regions for analysis were chosen as shown in Figure 1D. (**E**–**G**) Recovery of motor function of WT (*n* = 13) and *GFAP-C3a* (*n* = 12) mice assessed by changes in distance walked in the beam test between P2 and P7 (**E**), P7 and P14 (**F**), and P14 and P21 (**G**). Bar plots represent mean ± SEM. Two-way ANOVA with Šidák’s planned comparisons (**B** and **D**): *P < 0.05, ***P < 0.001, ****P < 0.0001 for ipsilesional vs. contralesional comparisons; ^#^*P* < 0.05, ^##^*P* < 0.01, ^###^*P* < 0.001 for between-genotype comparisons. Two-way ANOVA with repeated measures and Šidák’s planned comparisons (**E** and **G**): *P < 0.05.

To further examine the role of C3a/C3aR signaling in regulating reactive gliosis, we used mice that overexpress C3a in the injured CNS (*GFAP-C3a*) and their WT littermates. Infarct volume at P21 did not differ between the groups (*GFAP-C3a*, 2.15 ± 0.87 mm^3^, vs. WT, 1.58 ± 0.68 mm^3^, *P* = 0.085); however, GFAP expression in peri-infarct cortex was lower in *GFAP-C3a* mice (*P* < 0.001 for motor cortex and *P* < 0.05 for somatosensory cortex) and did not differ in the contralesional cortex (Figure 2, A and B). In *GFAP-C3a* mice, the density of Iba-1–positive cells was higher in peri-infarct motor and somatosensory cortex than in the contralesional hemisphere (*P* < 0.05 and *P* < 0.001, respectively; Figure 2, C and D). The density of Iba-1–positive cells in infarct-distal somatosensory cortex was higher in *GFAP-C3a* mice than in WT controls (*P* < 0.01; Figure 2D). These results further support a role for C3a/C3aR signaling in inhibiting astrocyte activation and stimulating microglia migration and/or proliferation in perilesional parenchyma.

The dynamics of motor function recovery differed substantially in *GFAP-C3a* mice and WT controls. Recovery started in the first week after stroke in WT mice (*P* < 0.05; Figure 2E) but not until the third week in *GFAP-C3a* mice (*P* < 0.05; Figure 2, F and G). In combination with the findings in the *C3aR^–/–^* mice, these results show that C3aR bidirectionally regulates glial responses in the postischemic brain and plays a dual role in functional recovery after ischemic brain injury. These results also suggest that the timing of potential interventions targeting C3aR is critically important.

### Reduced stroke-induced astrocyte reactivity in mice treated with intranasal C3a.

**Figure 3:**
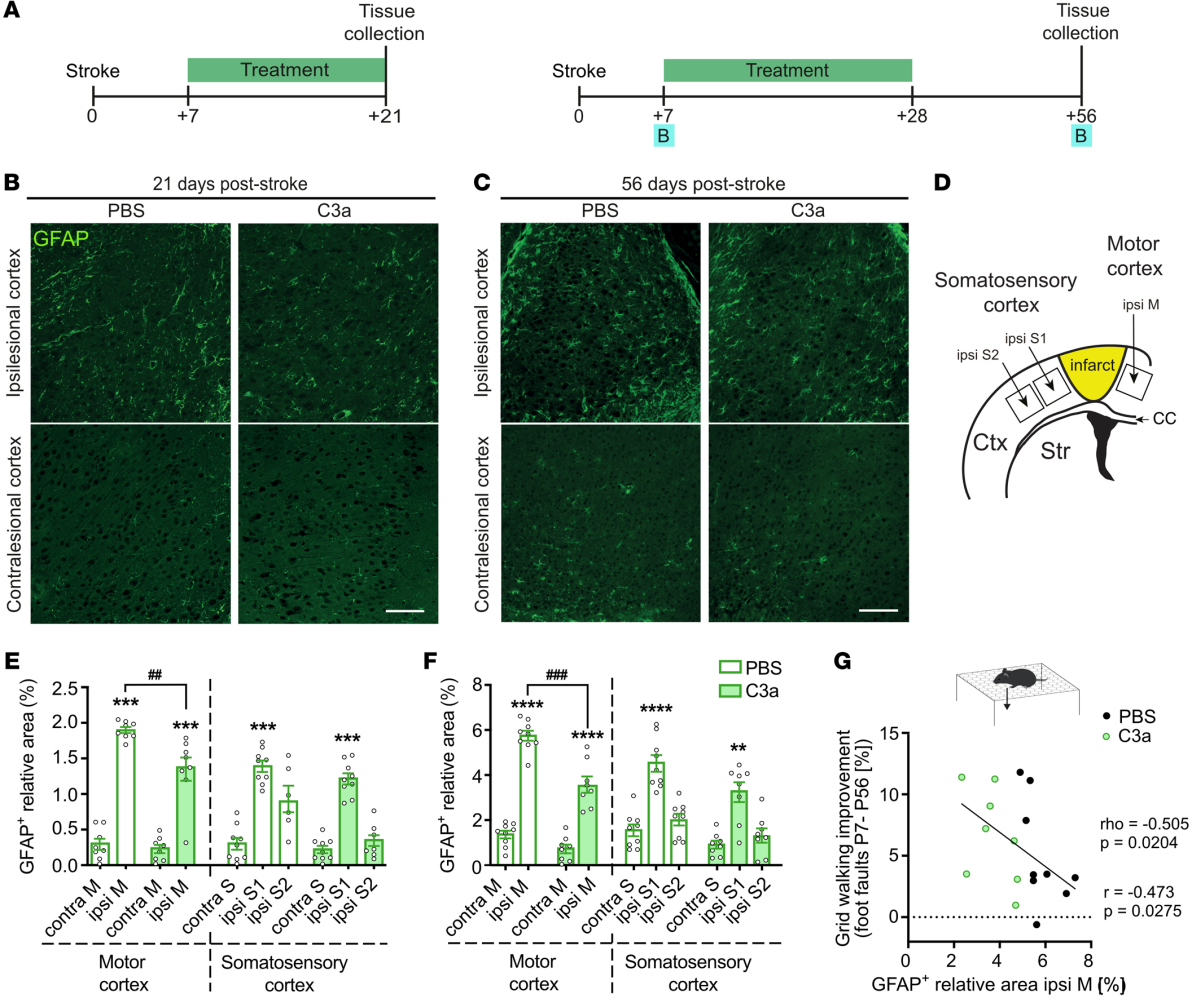
Intranasal C3a reduces astrocyte reactivity in peri-infarct cortex. (**A**) Study design. (**B** and **C**) Representative images of ipsilesional and contralesional cortex of mice treated with PBS or C3a. Astrocytes were visualized with antibodies against GFAP on P21 (**B**) and P56 (**C**). Scale bars: 100 μm. (**D**) Schematic of cortical regions chosen for analysis. Ctx, cortex; CC, corpus callosum; Str, striatum; ipsi, ipsilesional; M, motor cortex; S, somatosensory cortex. (**E** and **F**) Relative GFAP-positive area in proximal peri-infarct and contralesional cortex of mice treated with PBS or C3a on P21 (**E**) or P56 (**F**). PBS, *n* = 9; C3a, *n* = 8–9. (**G**) Association between GFAP expression in ipsilesional motor cortex on P56 and improvement in grid walk test, defined as the difference in percentage of right (affected) front paw foot faults, between P7 and P56. *r*, Pearson’s correlation coefficient; rho, Spearman’s correlation coefficient. Line represents the linear regression fit. Bar plots represent mean ± SEM. Two-way ANOVA with Šidák’s planned comparisons: **P < 0.01, ***P < 0.001, ****P < 0.0001 for ipsilesional vs. contralesional comparisons; ^##^*P* < 0.01, ^###^*P* < 0.001 for between-treatment comparisons.

Given the dual role of C3aR signaling and the positive effect of intranasal C3a treatment on functional recovery in the postacute phase after stroke (27), we assessed the effects of pharmacological modulation of C3aR signaling on peri-infarct astrocyte reactivity. To this end, WT mice were treated with intranasal C3a or PBS daily for 2 weeks, starting on P7, and GFAP expression was analyzed on P21. To examine long-term effects of the treatment, another cohort of mice was treated for 3 weeks, and reactive gliosis was determined 4 weeks later (P56) (Figure 3, A and D). At P21, we found increased GFAP expression in peri-infarct motor cortex and in peri-infarct somatosensory cortex of PBS-treated mice (Figure 3, B and E), as in the *C3aR^+/+^*, *C3aR^–/–^*, and WT mice in the aforementioned cohorts (Figure 1B and Figure 2B). In peri-infarct motor cortex, the relative GFAP-positive area was lower in C3a-treated mice than in PBS controls (*P* < 0.01; Figure 3, B and E), as in the *GFAP-C3a* mice (Figure 2B). At P56, GFAP expression was higher in both motor and somatosensory peri-infarct cortex than in contralesional cortex in both groups (Figure 3, C and F); the relative GFAP-positive area in the motor cortex was also lower in C3a-treated mice (*P* < 0.001; Figure 3F). Infarct volume did not differ between groups at P21 (PBS, 0.80 ± 0.21 mm^3^, vs. C3a, 0.89 ± 0.39 mm^3^, *P* = 0.429). Thus, astrocyte reactivity in peri-infarct cortex persists for at least 8 weeks after the ischemic event, and intranasal treatment with C3a starting on P7 reduces peri-infarct astrocyte reactivity. Remarkably, these effects persisted for at least 4 weeks after treatment cessation.

### Inverse correlation between GFAP expression in peri-infarct cortex and functional recovery.

Next, we examined a possible association between the extent of reactive gliosis in the peri-infarct cortex and functional recovery. Interrogating our previously published functional recovery data (27) for WT mice that received 3 weeks of daily intranasal treatment with C3a or PBS, we calculated the correlation between GFAP expression in peri-infarct cortex at 8 weeks (P56) after stroke and the improvement in motor performance between P7 and P56. We found that the relative GFAP-positive area in peri-infarct motor cortex correlated negatively with functional improvement in the grid walk test (Figure 3G). Thus, GFAP-expressing reactive astrocytes appear as important negative regulators of neuronal functioning in the chronic phase after stroke.

### No increase in microglia density in peri-infarct cortex of C3a-treated mice.

**Figure 4:**
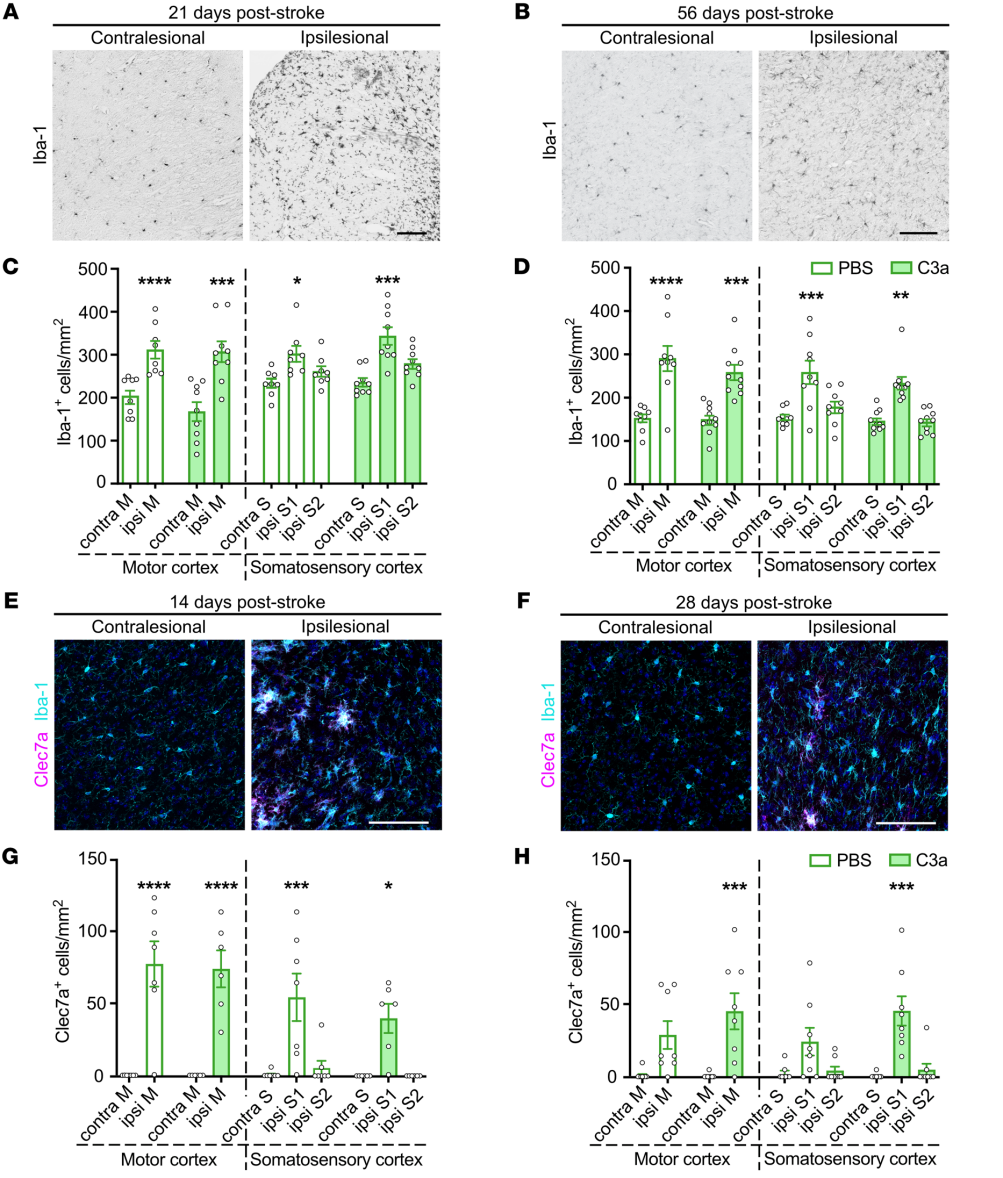
Intranasal C3a does not affect the density of microglia in peri-infarct cortex. (**A** and **B**) Representative images of contralesional and ipsilesional cortex stained with antibodies against Iba-1 on P21 (**A**) and P56 (**B**). Cortical regions were chosen for analysis as shown in Figure 3D. Scale bars: 100 μm. (**C** and **D**) Density of Iba-1–positive cells in the proximal peri-infarct and contralesional cortex of mice treated with PBS or C3a on P21 (**C**) or P56 (**D**). PBS, *n* = 8–9; C3a, *n* = 9–10. (**E** and **F**) Representative images of contralesional and ipsilesional motor cortex stained with antibodies against Iba-1 and Clec7a on P14 (**E**) and P28 (**F**). Cortical regions were chosen for analysis as shown in Figure 3D. Scale bars: 100 μm. (**G** and **H**) Density of Clec7a-positive cells in the proximal peri-infarct and contralesional cortex of mice treated with PBS or C3a on P14 (**G**) or P28 (**H**). P14: PBS, *n* = 6; C3a, *n* = 6. P28: PBS, *n* = 10; C3a, *n* = 10. Bar plots represent mean ± SEM. Contra, contralesional; ipsi, ipsilesional; M, motor cortex; S, somatosensory cortex. Two-way ANOVA with Šidák’s planned comparisons: *P < 0.05, **P < 0.01, ***P < 0.001, ****P < 0.0001 for ipsilesional vs. contralesional comparisons.

Next, we assessed the density of Iba-1–positive cells in peri-infarct cortex of the short- and long-term cohorts of C3a-treated mice. At both P21 and P56, the density of Iba-1–positive cells was significantly higher in peri-infarct cortex than in the contralesional hemisphere, with no differences between C3a-treated mice and PBS controls (Figure 4, A–D). To determine the effect of C3a treatment on the phenotype of peri-infarct microglia, we quantified the expression of C1q, a marker of neurotoxic microglia (10), and C-type lectin domain containing 7A (Clec7a), a marker of disease-associated microglia (35), at P14 (after 7 days of treatment) and P28 (after 21 days of treatment). At both P14 and P28, C1q immunoreactivity was higher in peri-infarct cortex than in the contralesional hemisphere, with no difference between the treatment groups (Supplemental Figure 1). At P14, 15% ± 3.9% and 18% ± 2.6% of Iba-1–positive cells in the peri-infarct motor cortex expressed Clec7a in the PBS- and C3a-treated mice, respectively; in both groups the density of Clec7a-positive cells was higher than in the contralesional hemisphere. At P28, the density of Clec7a-positive cells in the peri-infarct cortex was increased only in C3a-treated mice, with 23% ± 8.7% of Iba-1–positive cells expressing Clec7a (Figure 4, E–H). Intranasal treatment with C3a starting on P7 does not affect microglia density or C1q expression in peri-infarct cortex but may extend the time interval during which these cells exhibit the disease-associated phenotype.

Since C3a/C3aR signaling regulates endothelial VCAM1 expression associated with blood-brain barrier dysfunction in aged mice (32), we next examined the effects of C3a treatment on the expression of VCAM1. While we detected abundant CD31-positive endothelial cells expressing VCAM1 on sections of mouse spinal cord with motor neuron disease, we did not detect any such VCAM1-positive endothelial cells in the brain of C3a- or PBS-treated mice 28 days after stroke (Supplemental Figure 2). Thus, in non-aged mice neither ischemic stroke nor C3a treatment leads to endothelial cell activation.

### Altered stroke-induced responses of peri-infarct astrocytes in C3a-treated mice.

**Figure 5:**
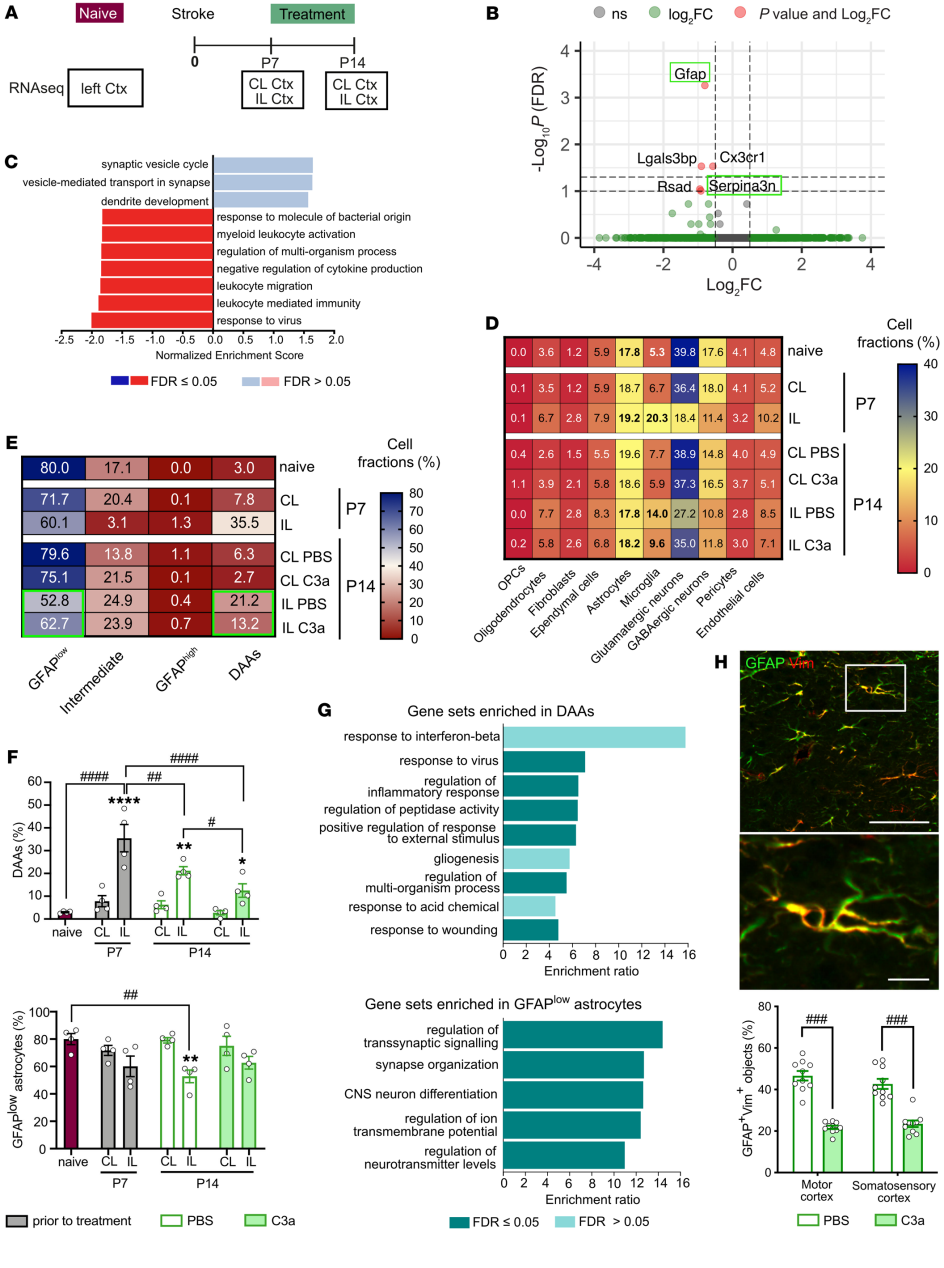
C3a treatment modulates stroke-induced astrocyte responses in peri-infarct cortex. (**A**) Experimental design. Ctx, cortex; CL, contralesional; IL, ipsilesional. (**B**) Volcano plot showing genes differentially expressed (adjusted *P* value < 0.1) in peri-infarct cortex of C3a- versus PBS-treated mice on P14. Green boxes indicate reactivity markers characteristic of DAAs. (**C**) Gene set enrichment analysis of differentially expressed genes in peri-infarct cortex of C3a- versus PBS-treated mice at P14. (**D**) Heatmap of cell type fractions estimated by deconvolution analysis. (**E**) Heatmap of astrocyte subpopulation fractions estimated by deconvolution analysis. Values in heatmap cells are group averages. (**F**) Sample variance and statistical analysis for the estimated contribution of the DAA and GFAP^lo^ subpopulations. *n* = 4 per group and time point. (**G**) Gene ontologies for the most highly expressed genes (gene expression profile score > 90 counts per million) in DAAs (top) and GFAP^lo^ astrocytes (bottom). FDR, false discovery rate. (**H**) Representative images of P21 peri-infarct cortex immunostained with antibodies against GFAP and vimentin (Vim) and the fraction of GFAP-positive astrocytes with overlapping Vim immunoreactivity in PBS- and C3a-treated mice on P21. PBS, *n* = 10; C3a, *n* = 10. Scale bars: 50 μm (upper), 10 μm (lower). Bar plots represent mean ± SEM. Two-way ANOVA with Holm-Šidák post hoc test (**F** and **H**): *P < 0.05, **P < 0.01, ****P < 0.0001 for IL vs. CL comparisons; ^#^*P* < 0.05, ^##^*P* < 0.01, ^###^*P* < 0.001, ^####^*P* < 0.0001 for comparisons between treatments and time points.

To determine the effects of C3a treatment on gene expression in peri-infarct cortex, we used bulk RNA sequencing (RNA-Seq) on tissue collected from WT mice on P7 and P14 and corresponding cortical tissue from naive WT mice (Figure 5A). We found that after 7 days of daily treatment (P14), the astrocyte reactivity markers *Gfap* and *Serpina3n* and the microglial marker *Cx3cr1* were differentially expressed in peri-infarct cortex of PBS- and C3a-treated mice (Figure 5B). Functional enrichment analysis revealed in the C3a-treated group downregulation of genes involved in inflammatory processes and a trend toward upregulation of genes involved in synaptic function (Figure 5C).

Because *Gfap* expression differed most between C3a- and PBS-treated mice (Figure 5B), we applied cellular deconvolution analysis using a published single-nucleus RNA data set (36) to examine the effects of stroke and C3a treatment on individual cell types and on astrocyte subpopulations. The deconvolution analysis estimates the relative proportions of cell types in the bulk RNA-Seq sample, using a known reference gene expression profile for individual cell populations (Supplemental Figure 3A). We found an increase in the microglial fraction in peri-infarct cortex at P7 and P14 (*P* < 0.001 and *P* < 0.01, respectively, vs. naive mice; and *P* < 0.0001 and *P* < 0.05, respectively, vs. contralesional cortex); this response was mitigated in C3a-treated mice (Figure 5D and Supplemental Figure 3B). The relative contribution of astrocytes was not altered by stroke or by C3a treatment (Figure 5D and Supplemental Figure 3C). However, a substantial subpopulation of cells showed characteristics of disease-associated astrocytes (DAAs), originally described in a mouse model of Alzheimer’s disease, aged mice, and aging human brain (36), in peri-infarct cortex on P7 and P14 (*P* < 0.0001 and *P* < 0.05, respectively, vs. naive mice; and *P* < 0.0001 and *P* < 0.05, respectively, vs. contralesional cortex). This DAA or DAA-like subpopulation, characterized by high expression of *Gfap*, *Vim*, and *C3ar1* (Supplemental Figure 3, D–F), was reduced in C3a-treated mice (*P* < 0.05). The subpopulation of homeostatic *Gfap*^lo^ astrocytes (36) was reduced on P14 only in PBS-treated mice (*P* < 0.01; Figure 5, E and F). The expression profile of the DAA fraction in peri-infarct cortex was enriched in genes that regulate the inflammatory response and the responses to virus and wounding, whereas the expression profile of the *Gfap*^lo^ astrocyte fraction was enriched in genes involved in neural plasticity (Figure 5G and Supplemental Figure 3, F and G). On brain sections from the 2-week treatment cohort, the fraction of GFAP-positive cells that coexpressed vimentin was reduced in peri-infarct cortex of C3a-treated mice (*P* < 0.001; Figure 5H), while the overall immunoreactivity of vimentin was not altered (Supplemental Figure 3H).

These results show that stroke leads to the appearance of *Gfap*^hi^ DAAs or DAA-like cells in the peri-infarct region, suggesting GFAP as a highly useful marker of this astrocyte subtype in postischemic cortex, and provide detailed insight into the effects of C3a treatment on astrocyte responses to ischemic injury.

### Increased structural connectivity in the post-stroke cortex of C3a-treated mice.

**Figure 6:**
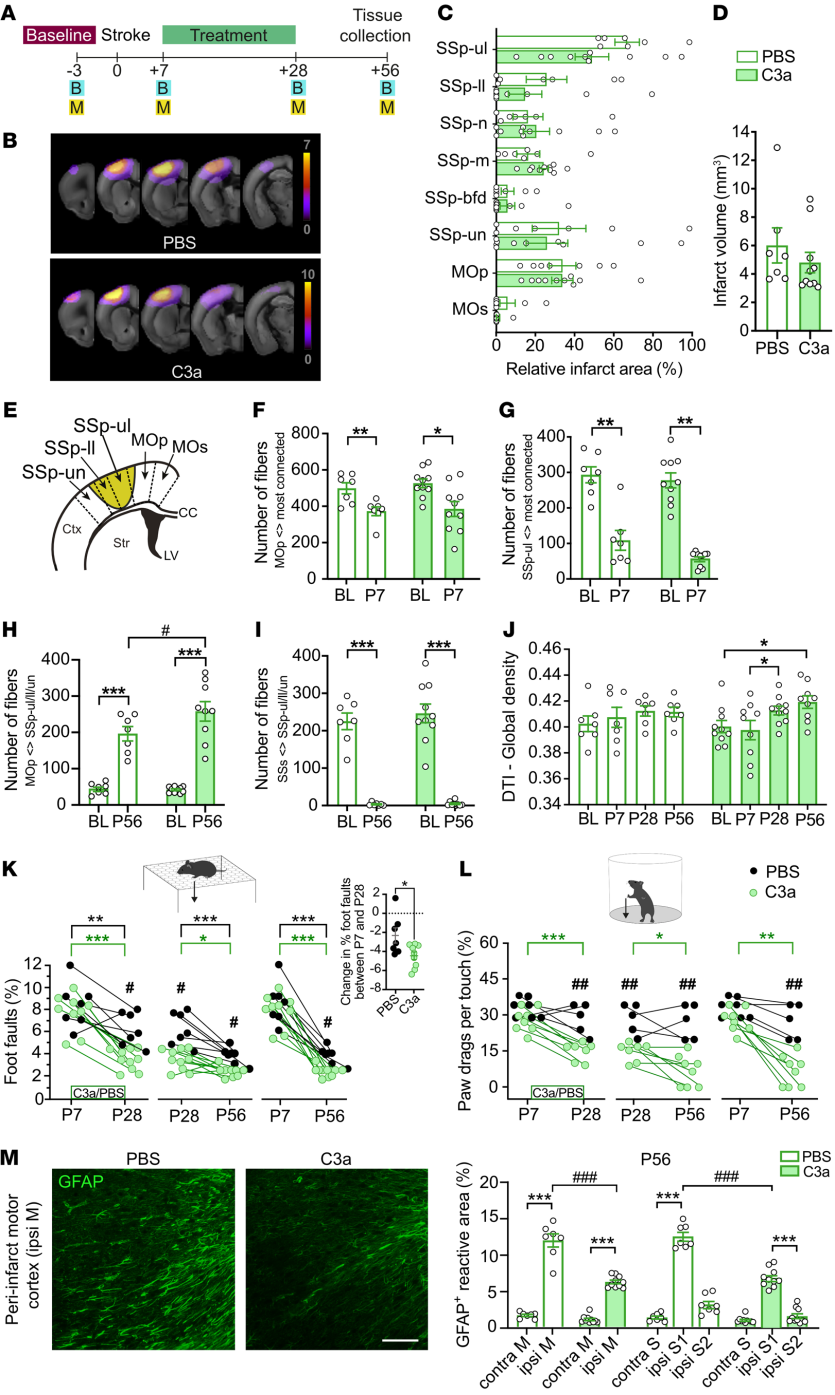
Intranasal C3a modulates post-stroke cortical connectivity. (**A**) Study design. (**B**) 3D illustration of T2-weighted MRI (T2w-MRI) with the infarct (in red) and stroke incidence maps of PBS- and C3a-treated mice. (**C**) Infarct location based on quantitative lesion mapping using T2w-MRI: primary somatosensory area (SSp) upper limb (SSp-ul), lower limb (SSp-ll), nose (SSp-n), mouth (SSp-m), barrel field (SSp-bfd), unassigned (SSp-un); primary and secondary motor area (MOp and MOs). (**D**) Infarct volume based on quantitative lesion mapping using T2w-MRI on P7. (**E**) Regions selected for fiber tracking analysis. (**F** and **G**) Number of fibers from ipsilesional MOp (**F**) and SSp-ul (**G**) to the most strongly connected regions before stroke (BL) and on P7. (**H** and **I**) Number of fibers from ipsilesional MOp (**H**) and supplemental somatosensory area (SSs) (**I**) to primary somatosensory cortex before stroke (BL) and on P56. (**J**) DTI global density (ratio of connections to the maximum possible number of connections for all 96 brain regions) before stroke (BL) and on P7, P28, and P56. (**K** and **L**) Recovery of motor function as assessed by change in the frequency of foot faults in the grid walk test (**K**) and paw drags per touch in the cylinder test (**L**) between P7 and P56. (**M**) Representative images of ipsilesional motor cortex of PBS- and C3a-treated mice in which astrocytes are visualized with antibodies against GFAP on P56. Scale bar: 100 μm. Relative GFAP-positive area in proximal peri-infarct and contralesional cortex of PBS- and C3a-treated mice (P56). PBS, *n* = 7; C3a, *n* = 10. Bar plots represent mean ± SEM. Two-way mixed effects analysis with false discovery rate correction (**F**–**J**); 2-way mixed effects analysis with Šidák’s corrections (**K** and **L**); 2-way ANOVA with Šidák’s planned comparisons (**M**); *P < 0.05, **P < 0.01, ***P < 0.001 for comparison between time points or ipsilesional vs. contralesional; ^#^*P* < 0.05, ^##^*P* < 0.01, ^###^*P* < 0.001 for between-treatment comparison.

Degeneration and regeneration of white matter have been proposed as important mechanisms of sensorimotor deficits and recovery after clinical and experimental stroke (37, 38). Water diffusion properties of tissue measured by in vivo diffusion tensor imaging (DTI) correlate with microstructural integrity and white matter density (39, 40). The integrity of the corticospinal tract, as determined by DTI in the acute phase, and diffusion properties in intrahemispheric primary, premotor, and supplemental motor cortex tracts predict long-term motor function outcome (41, 42). Since C3aR signaling helps regulate peri-infarct *Gfap* expression, which is associated with expression of neural plasticity–related genes (Figure 5G) and functional improvement (Figure 3G), we next sought to assess the effects of C3a treatment on stroke-induced changes in structural connectivity. To this end, we used DTI in a separate cohort of WT mice that were subjected to ischemic stroke and treated intranasally with C3a or PBS for 3 weeks (Figure 6A). We found that the 2 treatment groups did not differ in lesion location, infarct volume (Figure 6, B–D), or loss of fibers, indicative of loss of neuronal connections of the injured somatosensory and motor cortex in the first 7 days after stroke induction (Figure 6, E–G). However, on P56 structural connectivity between peri-infarct primary motor cortex and somatosensory cortex was 31% greater in C3a-treated mice (*P* < 0.05; Figure 6H). In more distant brain regions that were not directly affected by stroke (e.g., secondary somatosensory cortex), both C3a- and PBS-treated mice showed a pronounced loss of connectivity to stroke-affected regions (*P* < 0.001; Figure 6I). Moreover, in C3a-treated but not PBS-treated mice, DTI global density — a measure of overall fiber density in the whole brain indicative of white matter reorganization — was greater on P28 (i.e., at the end of the treatment) than at P7 and on P56 than at baseline (*P* < 0.05; Figure 6J).

C3a-treated mice had better and faster recovery of motor function between P7 and P56 as assessed by the grid walk and cylinder tests (Figure 6, K and L) and were less impaired both on P28 (the end of treatment) and on P56. As in the previous 2 cohorts of C3a-treated mice (Figure 3, E and F), GFAP immunoreactivity in peri-infarct cortex was persistently lower in C3a-treated mice in this cohort (Figure 6M).

Reactive astrocytes in the perilesional cortex extend their processes radially in the direction of the lesion (Figure 6M). This alignment of astrocyte processes leads to increased DTI-based fractional anisotropy, an indirect in vivo measure of astrocyte reactivity, and consequently may artifactually influence the determination of fiber tracts (43). Similarly, higher neuronal fiber density will increase fractional anisotropy (43). Accumulation and activation of microglia and macrophages can also lead to changes in fractional anisotropy (44). We therefore determined fractional anisotropy in the peri-infarct region as well as the stroke-affected and corresponding contralesional cortical areas (Supplemental Figure 4, A–C). We found that stroke led to a long-lasting increase in fractional anisotropy in peri-infarct cortex, with no difference between groups (Supplemental Figure 4, D and G). Other diffusion measures were also unaffected by C3a treatment (Supplemental Figure 4, E and F). Peri-infarct GFAP expression did not correlate with fractional anisotropy (*r* = 0.276, *P* = 0.30). Since gliosis beyond the direct vicinity of the peri-infarct glial scar is very limited at P56, it is unlikely that the gliosis-related changes in fractional anisotropy significantly affected or distorted the peri-infarct fiber count assessment. However, the increase in neuronal fiber density in the peri-infarct cortex may have reduced the net effect of C3a treatment on fractional anisotropy. Together, the comparable fractional anisotropy and peri-infarct Iba-1 immunoreactivity in PBS- and C3a-treated mice, and the reduced expression of GFAP and increased fiber tract density in C3a-treated mice, support the conclusion that intranasal treatment with C3a in the postacute phase after stroke promotes functional recovery at least in part by modulating neuronal connectivity and astrocyte reactivity.

### Increased expression of Igf1 and Thbs4, positive regulators of neural plasticity, in peri-infarct cortex of C3a-treated mice.

**Figure 7:**
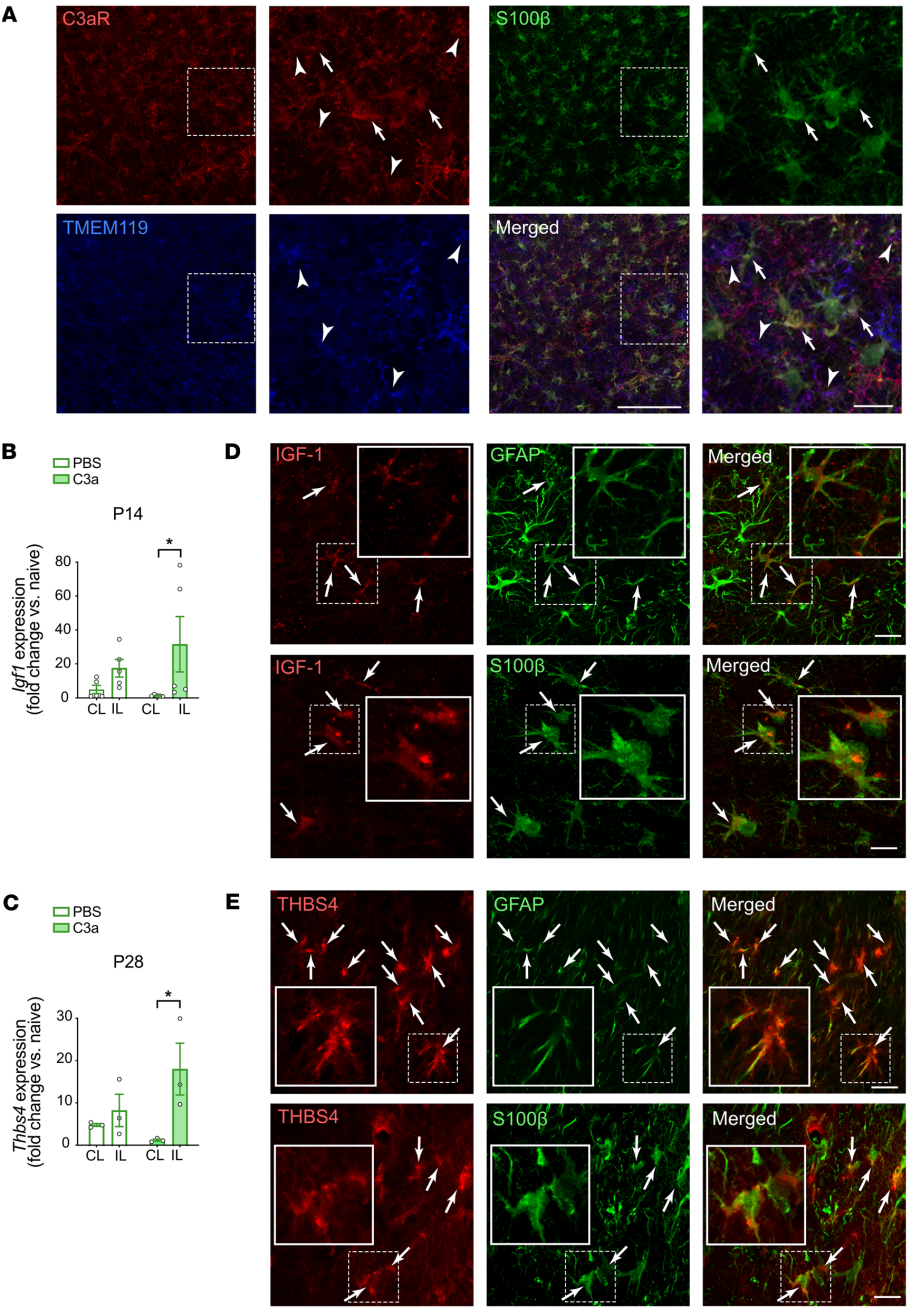
Intranasal C3a treatment upregulates the expression of genes encoding IGF-1 and THBS4 in peri-infarct cortex. (**A**) C3aR is expressed by astrocytes (arrows) and microglia (arrowheads) in the peri-infarct region on P7. Astrocytes are visualized by antibody against S100β; microglia are visualized by antibody against TMEM119. Scale bars: 100 μm (left), 20 μm (right). (**B**) Relative expression of *Igf1* in peri-infarct and contralesional cortex of mice treated with PBS or C3a for 7 days starting on P7. Bar plots represent mean ± SEM. PBS, *n* = 5; C3a, *n* = 5. (**C**) Relative expression of *Thbs4* in peri-infarct and contralesional cortex of mice treated with PBS or C3a for 21 days starting on P7. PBS, *n* = 3; C3a, *n* = 3. Bar plots represent mean ± SEM. Two-way ANOVA with Šidák’s planned comparisons: *P < 0.05. (**D** and **E**) IGF-1 (**D**) and THBS4 (**E**) immunoreactivity (arrows) within and in the vicinity of astrocytes visualized by antibodies against GFAP or S100β in peri-infarct cortex on P7 (IGF-1) or P28 (THBS4). Insets show higher magnification images of cells delineated by dashed squares. Scale bars: 20 μm (main), 10 μm (insets).

On P7, C3aR was expressed by peri-infarct TMEM119-positive microglia and S100β-positive astrocytes (Figure 7A) and to a lesser extent by neurons and endothelial cells (Supplemental Figure 5). Quantitative reverse transcriptase PCR showed a trend toward higher *C3ar1* mRNA levels in peri-infarct than in contralesional cortex (10.63 ± 3.83 vs. 1.09 ± 0.71, *P* = 0.052). On P7, P14, and P28, we measured the mRNA levels of genes that help regulate neural plasticity and are expressed in microglia and astrocytes (Supplemental Figure 6, A–C). We found that expression of *Igf1*, which encodes insulin-like growth factor 1 (IGF-1), was higher in peri-infarct cortex than in contralesional cortex of C3a-treated but not PBS-treated mice on P14 (*P* < 0.05; Figure 7B). In PBS-treated but not C3a-treated mice, stroke reduced the expression of *Bdnf*, which encodes brain-derived neurotrophic factor (Supplemental Figure 6C). Expression of *Thbs4*, which encodes thrombospondin-4 (THBS4), was increased in peri-infarct cortex of C3a- but not PBS-treated mice on P28 (Figure 7C). These effects were absent in *C3aR^–/–^* mice (Supplemental Figure 6, D–F). Immunostaining of brain sections revealed that in the peri-infarct cortex, IGF-1 and THBS4 were expressed predominantly by astrocytes (Figure 7, D and E, and Supplemental Figures 7 and 8), and THBS4 often appeared as deposits in the vicinity of astrocytes (Figure 7E). Notably, expression of GFAP and expression of THBS4 in individual cells were inversely correlated (*r* = –0.3792, *P* < 0.01). The effects of C3a treatment on neuronal connectivity may therefore be mediated by increased expression of positive regulators of neural plasticity.

## Discussion

This study shows that C3aR has a dual role in functional recovery after ischemic stroke: it facilitates recruitment of inflammatory cells in the acute phase and modulates reactive gliosis in the later stages. The improved functional recovery of mice that received intranasal C3a in the postacute phase was accompanied by global white matter reorganization, increased cortical structural connectivity, and altered astrocyte reactivity in peri-infarct cortex.

The distinct effects of loss and gain of function of C3aR signaling on the dynamics of motor function recovery in our study provide evidence that C3aR has a dual role in postischemic brain. In the acute phase, C3aR signaling inhibits recovery, likely by recruiting circulating leukocytes, whereas in later stages it promotes functional improvement by stimulating neural plasticity (27) and white matter reorganization through direct effects on the neuronal compartment and/or indirectly through its effects on reactive gliosis. We hypothesize that the timing of any C3aR-targeting intervention is critical for the outcome: delayed initiation of C3a treatment enables the beneficial effects on astrocytes and neural plasticity without the unwanted recruitment of inflammatory cells and microglia.

C3aR is expressed by microglia (45), and C3a modulates microglial phagocytosis (46). C3aR also mediates the recruitment of monocytes into prefrontal cortex in mice subjected to chronic stress (47) and helps recruit leukocytes from the peripheral circulation to the brain parenchyma during inflammation (48). Systemic pretreatment with a C3aR antagonist reduces granulocyte infiltration and neurological impairment after ischemic stroke (49, 50). Thus, our finding that constitutive C3aR deficiency and C3a overexpression have opposite effects on functional recovery in the first week after ischemia likely reflects the role of C3aR signaling in leukocyte infiltration. Further, although reactive microgliosis in peri-infarct cortex persisted for at least 8 weeks, intranasal treatment with C3a did not affect microglia/macrophage density in peri-infarct cortex. Since monocyte-derived macrophages do not persist in the peri-infarct region beyond P8 (34), the effects of genetic manipulation of the C3a/C3aR axis on the peri-infarct density of Iba-1–positive cells 3 weeks after stroke were likely mediated predominantly by microglia migration and/or proliferation in the acute phase. This possibility is further supported by the distinct effects of C3a on Iba-1–positive cell density in C3a-overexpressing mice versus mice that received intranasal C3a treatment starting on P7 (i.e., 4 days after monocyte infiltration peaks) (51).

C1q mRNA is highly expressed by reactive microglia (52), and microglial C1q is a major regulator of astrocyte functions (10, 53, 54). Our results support previous findings of increased C1q protein levels in the ipsilesional cortex after ischemic stroke (55) and show that C1q does not act as a mediator of the effects of C3a treatment in the post-stroke brain. Clec7a is a marker of disease-associated microglia with increased phagocytic activity and the potential to restrict neurodegeneration (35, 56). Since our data indicate that C3a treatment may extend the time interval during which microglia exhibit the disease-associated phenotype, the potential modulation of microglial functions in the post-stroke brain by C3a merits further investigation.

In line with our previous results in untreated WT mice (57, 58), we found that acute loss of neuronal fibers in stroke-affected cortex was still detectable at P56 in the PBS-treated group. The increase in DTI-detectable density of neuronal fiber tracts in C3a-treated mice, both globally and in tracts specific to intrahemispheric sensorimotor connections, strongly suggests that C3a stimulates stroke-induced white matter reorganization. Since sensorimotor function improved faster in C3a-treated mice, increased global connectivity, and specifically intrahemispheric connectivity, could be an important contributor to functional recovery after stroke and can be enhanced by intranasal C3a administration.

In postischemic brain, astrocytes perform many functions that are important for remodeling of neural tissue and peri-infarct networks. These cells stimulate post-stroke synaptogenesis (59) and play a role in phagocytic clearance and brain remodeling (60). We reported that reduced GFAP expression in C3a-treated cultured astrocytes subjected to ischemic stress was associated with their increased survival and that C3a did not promote survival of *C3aR^–/–^* astrocytes (24). Combining a loss-of-function and a gain-of-function approach, we showed that signaling through the C3aR downregulates the expression of GFAP in peri-infarct tissue. Together, these results suggest that C3aR signaling modulates the function of astrocytes without compromising their survival. In addition, GFAP expression at both the protein and the mRNA level appears to be a robust measure of astrocyte reactivity in postischemic brain.

Using a pharmacological approach, we found that daily intranasal treatment of WT mice with C3a for 2 weeks starting on P7 had a modulatory effect on peri-infarct astrocyte reactivity that was similar to the effect of C3a overexpression. Moreover, astrocyte reactivity in peri-infarct cortex persisted for at least 8 weeks, and WT mice that received daily intranasal treatment with C3a for 3 weeks starting on P7 had reduced peri-infarct astrocyte reactivity 4 weeks after treatment cessation. At this time point, GFAP expression in peri-infarct cortex correlated inversely with motor function improvement. Astrocyte activation and glial scar formation are required for axonal regeneration in the injured spinal cord (61) and have an important neuroprotective role in the acute phase after stroke (12). Nevertheless, reduced astrocyte reactivity/GFAP expression in peri-infarct cortex 7 days after ischemic stroke is associated with increased axonal sprouting and better functional recovery (62), consistent with the association we found between functional improvement and reduced peri-infarct astrocyte reactivity. Delayed intranasal treatment with C3aR agonists appears to modulate late-phase astrocyte reactivity without interfering with the neuroprotective aspects of reactive gliosis and lesion demarcation in the acute phase.

DAAs have been identified near amyloid plaques in a mouse model of Alzheimer’s disease and in aged brain (36). Our results provide evidence that DAA-like cells are present in the post-stroke brains of young mice and suggest that this astrocyte state is not only amyloid-associated but is more universal. Our findings also suggest that such cells help regulate inflammation and tissue repair in the post-stroke brain, while homeostatic *Gfap*^lo^ astrocytes seem to be involved in neural plasticity. Notably, the relative abundance of these astrocyte subpopulations was affected by C3a treatment. Although the specific activities of DAAs in the different phases after ischemia are unknown, our findings suggest that C3aR is an attractive target to dampen the negative responses of DAAs in the post-stroke brain.

Previously, we proposed that modulation of reactive gliosis in peri-infarct cortex, particularly in the postacute phase, is a mechanistic link between neural plasticity–promoting pharmacological interventions and functional recovery after ischemic stroke (4, 14). Here, we found that the modulatory effect of C3a on peri-infarct astrocyte reactivity is associated with upregulation of *Igf1* and *Thbs4*. IGF-1, expressed in the CNS by microglia/macrophages, astrocytes, and neurons (54, 63), enhances axon outgrowth of corticospinal motor neurons in vitro and in the developing CNS (64), and high serum IGF-1 levels just after the onset of ischemic stroke are associated with better neurological recovery in humans (65). IGF-1 expression is increased in peri-infarct astrocytes (66). *Igf1* expression in the lungs is positively regulated by C3aR (67). THBS4 is an astrocyte-derived protein (68, 69) that promotes excitatory synaptogenesis (70), and the expression of *Thbs4* increases after injury (68, 71). In line with a previous study (66), we found that *Igf1* is upregulated in peri-infarct cortex on P7. Although the expression of C3aR in the brain is not limited to astrocytes and the positive effects of C3a on post-stroke recovery conceivably involve also direct modulation of the functions of neurons and microglia, the increased expression of *Igf1* and *Thbs4* in the peri-infarct cortex of C3a-treated mice points to IGF-1 and THBS4 as glia-derived mediators of the effects of C3a/C3aR signaling on neuronal connectivity after stroke.

We acknowledge the use of only non-aged male mice as a limitation of our study. Given that sex and aging affect stroke incidence and outcome (72), and C3a/C3aR signaling mediates vascular inflammation and blood-brain barrier dysfunction during aging (32), further investigations are needed to determine the relevance of our findings in females and in aged mice. The differences in brain size and in the distance from the nasal cavity to the cerebral cortex between mice and humans may limit direct inference of our findings to clinical stroke. This issue needs to be addressed by future studies using larger animals and in clinical studies. Reports showing that in humans, intranasally administered insulin (51 amino acids) bypasses the bloodstream and reaches the cerebrospinal fluid within 30 minutes (73), modulates neuronal function (74), and reduces Alzheimer’s disease–associated changes in white matter (75) support the feasibility of using the nasal route for direct delivery of therapeutic peptides to the brain.

In summary, our results highlight some important features of ischemic injury–induced reactive gliosis and identify C3aR as a therapeutically relevant target to modulate glial responses after stroke. C3a treatment within a clinically convenient therapeutic window takes advantage of positive effects of C3a on astrocytes, white matter reorganization, and neuronal connectivity while avoiding the detrimental consequences of C3aR signaling during the acute phase and accelerating functional recovery. These findings provide what we believe to be novel opportunities for experimental modulation of astrocyte reactivity and for development of effective recovery-promoting strategies for stroke and possibly other neurological disorders as well.

## Methods

The detailed description of the materials and methods used is provided in Supplemental Methods.

### Data and code availability.

Raw and processed MRI data are available in the online repository GIN (https://doi.org/10.12751/g-node.699mgv). The MRI post-processing protocols and the MRI processing software AIDAmri and AIDAconnect are available from GitHub (https://github.com/aswendt-lab; commit IDs 62f0f89 and 245230c). Raw and processed RNA-Seq data are available through the NCBI’s Gene Expression Omnibus database (GEO GSE184917). Parameters for a standard gene expression and deconvolution analysis are provided in Supplemental Methods (no original code was generated).

### Study approval.

The protocols were approved by the Animal Ethics Committee of Gothenburg, Sweden (permits **(**146-2008, 170-2009, 308-2012, 41-2015, 2735-2020) and the Landesamt für Natur, Umwelt und Verbraucherschutz North Rhine–Westphalia, Germany (animal protocol 84-02.04.2014.A305).

## Author contributions

AS, M Pekna, MH, and M Pekny conceived the study. AS, MA, DZ, SL, FW, JMS, ALA, YL, MM, JL, DW, MD, ÅTN, PA, and LV acquired and analyzed data. AS, MA, DZ, SL, DW, MD, PA, LV, MK, MH, M Pekna, and M Pekny interpreted data. AS, M Pekna, MA, MK, LV, and M Pekny acquired funding. AS, M Pekna, and M Pekny wrote the manuscript. All authors read, edited, and approved the final manuscript.

## Supplementary Material

Supplemental data

## Version 1 03/30/2023

In-Press Preview

## Version 2 05/15/2023

Electronic publication
