# Supplementary material for: Complement C3a treatment accelerates recovery after stroke via modulation of astrocyte reactivity and cortical connectivity
Source: J Clin Invest. 2023 May 15;133(10):e162253. doi: 10.1172/JCI162253 (PMC10178843; doi:10.1172/JCI162253)
Supplement: Supplemental data [file jci-133-162253-s172.pdf]

Supplemental materials for

Complement C3a treatment accelerates recovery after stroke via modulation of astrocyte reactivity and cortical connectivity

Anna Stokowska, Markus Aswendt, Daniel Žucha, Stephanie Lohmann, Frederique Wieters, Javier Morán Suarez, Alison L. Atkins, YiXian Li, Maria Miteva, Julia Lewin, Dirk Wiedermann, Michael Diedenhofen, Åsa Torinsson Naluai, Pavel Abaffy, Lukas Valihrach, Mikael Kubista, Mathias Hoehn, Milos Pekny, Marcela Pekna*

***Corresponding author**: marcela.pekna@neuro.gu.se

This PDF file includes:

Supplemental Methods

Supplemental Figures 1 to 8

Supplemental Methods

Mice

C3aR$^{-/-}$ mice (76) were backcrossed onto the C57BL/6 genetic background for 10 generations. Heterozygous mice were then intercrossed to generate homozygous *C3aR*$^{-/-}$ mice. WT C57BL/6NCr mice served as controls (*C3aR*$^{+/+}$). *GFAP-C3a* mice on a C57BL/6NCr genetic background were generated as described (77); WT littermates served as controls. Male 7–9-month-old mice weighing 35–45 g were used. For intranasal treatment experiments, male 5-month-old WT C57BL/6NCr mice (Charles River) weighing 30–35 g were used. For the MRI experiments, 8–10-week-old male WT mice (Charles River) weighing 22–27 g were used. Mice were housed under standard conditions on a 12 h light/12 h dark cycle with food and water *ad libitum*. All experiments were conducted in accordance with the standards for preclinical studies of stroke therapy (78) and followed the ARRIVE (Animal Research: Reporting of In Vivo Experiments) guidelines.

Photothrombotic stroke induction

Cortical photothrombosis was induced with the Rose Bengal method as described (27). Briefly, anesthesia was induced with 5% isoflurane (Forene, Abbott) in air and oxygen (1:1) and maintained with 2.5% isoflurane. Body temperature was monitored with a rectal probe and maintained at 37°C. Anesthetized mice were placed in a stereotaxic frame, and the skull was exposed through a midline scalp incision. Rose Bengal (200 µl, 10 mg/ml in sterile saline, Sigma) was injected intraperitoneally. After 5 min, the skull and underlying brain tissue were illuminated for 15 min with a 2-mm diameter cold laser beam (50 mW, 561 nm; Cobolt AB) positioned at AP 0.5 mm and ML –2.7 mm relative to the bregma, to target the border between primary somatosensory and motor cortex in the left hemisphere (79). In mice that received

intranasal treatment for 2 or 3 weeks (data presented in Figures 3, 4, 5, 7, S1 and S3), the protocol was modified to target the motor cortex as follows. Transcranial illumination was delivered for 15 min with cold light source (LQ1600, Fiberoptic-Heim AG) equipped with 2 mm-wide fiber optic probe and directed to AP 0 and ML –1.5 relative to the bregma. For the MRI study (data presented in Figure 6 and S2), photothrombosis was induced as described (58). In brief, 150 µl (1.5 mg) of Rose Bengal was injected intraperitoneally followed by 50 mW laser radiation at 561 nm for 15 min at brain coordinates AP 0.5 mm and ML –2.5 to target the primary somatosensory forelimb area and primary motor cortex. The scalp was then sutured, wound was infiltrated with bupivacaine (50 µl, 0.25%, Marcain, Astra Zeneca) and mice were placed in a warm cage for 45 min to recover from anesthesia. Upon return to their home cage, mice were given moist mashed food. Body weight was monitored daily for 7 days after surgery. The cohorts did not differ in body weight loss, body temperature during surgery (36.5 ± 0.5ºC), surgery duration, or surgery-related mortality (0-2%).

Intranasal treatment

The intranasal treatment was done as described (27). Purified human C3a (Complement Technologies) was diluted in sterile phosphate-buffered saline (PBS) to a concentration of 200 nM, and 20 µl (10 µl/nostril; corresponding to approximately 1.13 µg/kg body weight) of C3a solution or PBS was given intranasally to awake, hand-restrained mice, held in a supine position. Solutions were administered through a pipette tip, drop-wise in 5-µl portions at 1-min intervals to allow for absorption. C3a or PBS was given every 24 hours on post-stroke day (P) 7 to P21 (short-term study) or on P7 to P28 (long-term and MRI studies). Mice were assigned to C3a or PBS treatment by randomization stratified by body weight, and experimenters remained blinded for the treatment.

Behavioral assessment of motor function

Motor function of *C3aR*$^{-/-}$ and *GFAP-C3a* mice and controls was assessed by round beam walk test, in which mice traversed a 60 cm-long round metal beam 1 cm in diameter suspended 60 cm over the lab bench. A foam cushion was placed beneath the beam to soften the landing after falls. A cage similar to the home cage was placed at the end of the beam to encourage directional crossing. Mice were trained to complete the task 72–24 hours before stroke induction and were tested on P2, P7, P14, and P21. Distance traveled until the first fall or within a maximum time of 2 min was measured, and changes in performance over time after stroke were assessed for each group. Due to changes in animal and stroke induction protocols, motor function recovery of the intranasally treated mice was assessed by the cylinder and grid walk tests as described (58). Briefly, mice were habituated to the test environment and procedure 3 times within 2 weeks before stroke induction. Baseline assessment was done 3–4 days before stroke induction, followed by tests on P3, P7, P14, P28, P42, and P56. All tests were video recorded and analyzed to quantify paw foot faults and paw drags (58, 80). Motor function improvement in the grid walk test was determined as the difference between the percentage of front paw foot faults on P7 and P56 (Figure 3G) or difference between the percentage of front paw foot faults on P28 and P7 (Figure 6K, inset). Grid walking improvement data in Figure 3G are based on results that were presented in full in a previous publication (27). Investigators who performed the behavioral studies and analyzed data were blinded to genotype and treatment group.

Magnetic resonance imaging

MRI was done at the Max Planck Institute for Metabolism Research, Cologne, using a 94/20USR BioSpec Bruker system with the 660 mT/m B-GA12SHP gradient system, RT-shim and related power supplies, the 1H receive-only mouse brain surface coil, and the 1H resonator 112/072 – operated with ParaVision v6.0.1 (Bruker, BioSpin). To reduce movement artifacts and provide reproducible mouse brain placement, mice were anesthetized with isoflurane (2–

3% in 70/30 N_2/O_2) and head-fixed with tooth and ear bars in an animal carrier. Respiration and body temperature were measured with a custom-made system based on DASYLab (National Instruments) and electronically recorded (Small Animal Instruments). Body temperature was maintained at 37°C with a feedback-controlled water circulation system (medres Gmbh). After initial adjustments (RF power, shim, and B0 field), a high-resolution, whole-brain, T2-weighted RARE sequence (T2w-MRI) was acquired at coronal slice orientation with a RareFactor of 8, repetition time (TR) of 5,500 ms, an effective echo time (TE) of 32.5 ms, voxel size of 0.068 x 0.068 x 0.5 mm^3, field-of-view of 17.5 x 17.5 mm^2, a matrix size of 256 x 256 with 28 noncontiguous slices (0.3 mm gap), and a scan time of 5:52 min. With the same slice orientation, DTI was acquired with an 8-shot, spin-echo, echo-planar imaging (EPI) sequence with a b value of 670 s/mm^2, gradient duration of 3.5 ms, gradient separation of 8 ms, TR of 3,000 ms, TE of 17.5 ms, voxel size of 0.141 x 0.141 x 0.5 mm^3, field-of-view of 18 x 18 mm^2, matrix size of 128 x 128 with 20 slices, and acquisition time of 14 min.

Image pre- and postprocessing

For atlas-based lesion mapping and quantification of infarct size and location on P7 after stroke, we used a workflow based on the combination of T2-weighted MRI and the 3D reference atlas, Allen Mouse Brain Common Coordinate Framework, CCFv3 (81). T2-weighted and DTI data were pre-processed with our in-house Python pipeline AIDAmri (82), which included brain extraction, bias-field correction, and two-step image registration with the atlas (81). Stroke masks were semi-automatically segmented with the 3D snake evolution tool ITK-SNAP (83). Transformation from the registration atlas to T2-weighted MRI was applied to calculate the percentage of infarct per atlas region. Notably, the infarct volume data obtained on P7 reflect the primary lesion together with vasogenic edema.

DTI processing, deterministic fiber tracking, and calculation of diffusion measures fractional anisotropy, axial diffusivity, and radial diffusivity were done as described with the

in-house tools AIDAmri and DSI Studio (http://dsi-studio.labsolver.org; (57). DTI global density was calculated as the ratio of detected connections divided by the maximum possible number of connections between all 96 brain regions (i.e., with a global density of 1, all brain regions would be structurally connected with each other).

Individual peri-infarct regions of interest for the diffusion calculations in AIDAmri were generated with a workflow written in Python (v1.1, https://github.com/aswendtlab/Project_C3a_peri-infarct). Briefly, the peri-infarct area was defined as a dilation of the stroke mask by about 15 pixels, yielding an individual irregular ring-shaped border area around the stroke core at P7, using the SciPy function binary dilation. This area was aligned to the DTI data by applying the calculated co-registration parameters for individual time points and the template as well as the known relation between the scans (T2 vs. DTI). Template labels were transferred to the DTI space using the known relations and aligned in the same DTI image spaces. Peri-infarct regions of interest were created by including only pixels in the vicinity of the stroke core where template brain regions overlapped with the individual peri-infarct border area. As a result, cortical regions were replaced by the new peri-infarct-delimited regions for further analysis. Astrogliosis was quantified by diffusion MRI as described (43).

Tissue preparation and infarct volume quantification

Mice were deeply anesthetized with thiopental (Hospira) and transcardially perfused with 0.9% saline and then with 4% paraformaldehyde in 0.1 M PBS. Brains were removed and immersed in the same fixative overnight. Tissue was dehydrated, embedded in paraffin, and cut into 6-μm serial coronal sections. Every 20th section was stained with hematoxylin and eosin. Infarct size in the *C3aR*$^{-/-}$ mice, *GFAP-C3a* mice, their controls and intranasaly treated mice that did not undergo MRI was determined on digital images with ImageJ software (NIH, ver. 1.47q) by manual delineation of the infarct and hemisphere areas on sections spanning the entire lesion

along the anterior-posterior axis by an investigator blinded to experimental group. Infarct volume was calculated by multiplying area of total tissue loss including shrinkage due to scarring [(contralesional hemisphere - ipsilesional hemisphere) + infarcted tissue] on each section by the total distance between sections.

Immunostaining

After dewaxing, rehydration and 3 5-min rounds of heat-induced antigen retrieval with 0.01 M citrate buffer (pH 6, 0.05% Tween 20), sections were washed 3 times for 5 min each with 0.05% PBS-T (PBS containing 0.05% Tween 20) and incubated first with blocking buffer [4% normal donkey (Iba-1) or goat (GFAP) serum in PBS-T] for 1 hour at room temperature (RT) to reduce nonspecific protein binding and then with the primary antibody [rabbit anti-GFAP (Z0334, Dako; 1:200) or rabbit anti-Iba-1 (019-19741, Wako; 1:500) or a cocktail of guinea-pig anti-GFAP (#173 004, Synaptic Systems; 1:500) and rabbit anti-vimentin (ab92547, Abcam; 1:500)] in blocking buffer overnight at 4°C. One slide per staining batch was incubated with blocking buffer only as a negative control. Next, sections were washed 3 times for 5 min each with PBS-T and incubated with the secondary antibody [goat anti-rabbit Alexa Fluor 488 (GFAP; A11034, Invitrogen; 1:2000), biotinylated donkey anti-rabbit (Iba-1; 711-065-152, Jackson ImmunoResearch; 1:400) or a cocktail of goat anti-guinea-pig Alexa Fluor 488 (GFAP; A11073, Invitrogen; 1:500) and donkey anti-rabbit Alexa Fluor 555 (vimentin; A31572, Invitrogen; 1:500)] in blocking buffer for 1 hour at RT. Sections stained for GFAP or GFAP with vimentin were washed 3 times for 5 min each with PBS-T, mounted with ProLong Gold (P36931, Invitrogen), coverslipped, and sealed with nail polish 24 hours later. For Iba-1 staining, after incubation with the secondary antibody, sections were incubated with avidin/biotin-horseradish peroxidase complex (Vectastain Elite ABC kit, PK-6100, Vector Laboratories) followed by a diaminobenzidine Substrate Kit (SK-4100, Vector Laboratories)

according to the manufacturer's instructions. Next, sections were washed 3 times for 5 min each with PBS-T, dehydrated (70% EtOH 2 min, 95% EtOH 2 min, 100% EtOH 2 min), and cleared with xylene for 5 min. Slides were mounted with VectaMount medium (H-5000, Vector Laboratories) and coverslipped.

For C1q, Clec7a, VCAM1, C3aR,THBS4 and IGF-1 immunofluorescent labeling, brains from WT mice at P7, P14 or P28, and spinal cord from symptomatic *wobbler* mouse (84) as a positive control for the VCAM1 immunoreactivity were fixed as described above and immersed in 30% sucrose buffered with PBS at 4°C for cryoprotection until they sank in the solution. Free-floating 30-µm-thick coronal sections were washed 3 times for 5 min each in 0.1% PBS-T, blocked with 5% normal donkey serum or 5% normal goat serum in 0.3% PBS-T for 1 hour at RT, and incubated with rat anti-C1q antibody (ab11861, Abcam; 1:250) or a cocktail of primary antibodies in blocking buffer for 18-48 hours at 4°C. Following combinations of primary antibodies were used: rat anti-Clec7a (mabg-mdect, InvivoGen, 1:200) together with guinea pig anti-Iba-1 (HS-234308, Synaptic Systems, 1:500), rat anti-C3aR (mAb74, HyCult Biotech; 1:100) together with rabbit anti-S100β (Z0311, Dako; 1:200) and chicken anti-TMEM119 (#400 006, Synaptic Systems; 1:500), rat anti-C3aR with mouse anti-Map2 (M1406, Sigma; 1:200) or goat anti-podocalyxin (AF1556, R&D Systems; 1:500); goat anti-THBS4 (AF3290, R&D Systems; 1:100) together with rabbit anti-GFAP (Z0334, Dako; 1:1000), rabbit anti-S100β (Z0311, Dako; 1:200), guinea-pig anti-Iba-1 (HS 234 30; Synaptic Systems; 1:500), mouse anti-Map2 (M1406, Sigma; 1:200) or rabbit anti-Olig2 (13999-1-AP, Protein Tech; 1:200); goat anti-IGF-1 (AF791; R&D Systems; 1:1000) together with rabbit anti-GFAP (Z0334, Dako; 1:1000), rabbit anti-S100β (Z0311, Dako; 1:200), guinea-pig anti-Iba-1 (#HS 234 30; Synaptic Systems; 1:500), mouse anti-NeuN (MAB377, Millipore; 1:100) or rabbit anti-Olig2 (13999-1-AP, Protein Tech; 1:200). For VCAM1 immunolabeling, 30-µm-thick coronal brain sections or transverse spinal cord sections were washed 3 times for 5 min

each in 0.1% PBS-T, incubated with M.O.M mouse Ig blocking reagent (MKB-2213-1, VectorLabs) for 2 hours at RT, washed 3 times for 5 min each in 0.1% PBS-T, blocked with 5% normal donkey serum in 0.3% PBS-T for 1 hour at RT and incubated with mouse anti-VCAM1 (#919801, BioLegend; 1:500) and rabbit anti-CD31 (ab28364, Abcam, 1:50) primary antibodies in blocking buffer for 18 hours at 4°C.

The sections were then washed 3 times for 5 min each with 0.1% PBS-T and incubated with goat anti-rat Alexa Fluor 647 antibody (C1q; A21247, Invitrogen; 1:1000) or with a cocktail of secondary antibodies in blocking buffer for 2 hours at RT. Following combinations of secondary antibodies were used: goat-anti rat Alexa Fluor 647 (Clec7a, A21247, Invitrogen, 1:1000) together with donkey anti-guinea pig Alexa Fluor 488 (Iba-1, 706-545-148, Jackson ImmunoResearch, 1:1000), goat anti-rat Alexa Fluor 488 (C3aR; A11006, Invitrogen; 1:250) together with donkey anti-rabbit Alexa Fluor 555 (S100β; A31572, Invitrogen; 1:500) and goat anti-chicken Alexa Fluor 647 (TMEM119; A21449, Invitrogen; 1:250), together with donkey anti mouse Alexa Fluor 488 (NeuN; A21202, Invitrogen, 1:250) or with donkey anti-goat Alexa Fluor 555 (Podocalyxin; A21432, Invitrogen, 1:500); biotinylated donkey anti-goat (THBS4 or IGF-1; 705-065-147, Jackson ImmunoResearch; 1:400) together with donkey anti-rabbit Alexa Fluor 488 (GFAP, S100β or Olig2; A21206, Invitrogen; 1:500), with donkey anti-guinea pig Alexa Fluor 488 (Iba-1; 706-545-148, Jackson ImmunoResearch; 1:400), or with donkey anti-mouse Alexa Fluor 488 (Map2 and NeuN; A21202, Invitrogen, 1:250); donkey anti-mouse Alexa Fluor 555 (VCAM1; A31570, Invitrogen, 1:500) together with donkey anti-rabbit Alexa Fluor 488 (CD31; A21206, Invitrogen; 1:250). For C1q, C3aR, and VCAM1 immunolabelling, sections were then washed 3 time for 5 min each with 0.1% PBS-T, mounted with ProLong Gold (Invitrogen), coverslipped, and sealed with nail polish 24 hours later. For THBS4 and IGF-1 immunolabeling, after incubation with secondary antibodies, sections were washed 3 times for 5 min each with 0.1% PBS-T and incubated with an avidin/biotin-horseradish

peroxidase complex (Vectastain Elite ABC kit) for 1 hour at RT and then with Alexa Fluor 555-tyramide reagent (B40955, Invitrogen) according to the manufacturer's instructions. Finally, sections were washed, mounted, and coverslipped as above.

Image acquisition and analysis

Images from sections immunolabeled for GFAP or Iba-1 were analyzed with the Integrated Morphometry package in MetaMorph software (v7.8.6, Molecular Devices). The GFAP-positive relative area was quantified on single-plane confocal images obtained with an LSM 700 laser-scanning microscope (Carl Zeiss) or on epi-fluorescent images obtained with a Nikon Eclipse 80i equipped with an Axiocam 506 camera, (Carl Zeiss) and 20x objectives (NA 0.8 and 0.75, respectively). Iba-1-positive cell somata were counted on bright-field images obtained with a 20x objective (Nikon Eclipse 80i equipped with a Nikon DMX1200 camera or an Axiocam 506 camera, Carl Zeiss). Images were taken in the peri-infarct motor (M) and somatosensory (S) cortex within one or two adjacent microscopic fields of view around the infarct (excluding glial scar at the infarct border) and in the corresponding contralesional regions. Field of view location corresponded to the distance of 15–335 μm (proximal regions: ipsi M1 and ipsi S1) and 335–670 μm (distal regions: ipsi M2 and ipsi S2) from the infarct border for GFAP or 20–720 μm (proximal regions: ipsi M1 and ipsi S1) and 720–1420 μm (distal regions: ipsi M2 and ipsi S2) for Iba-1. Three sections 120 μm apart were analyzed per mouse. Data are presented as Iba-1 density (positive cells/mm^2) and as GFAP-positive area relative to the total image area (%).

GFAP and vimentin colocalization analysis was performed on images obtained with a Nikon Eclipse 80i equipped with an Axiocam 506 camera, (Carl Zeiss) and 20x objective using Colocalization application module within MetaMorph software (v7.8.6, Molecular Devices). Following manual delineation of the region of interest (ROI) within proximal peri-infarct motor (ipsi M) and somatosensory (ipsi S1) cortices, fluorescent signal in each channel was

thresholded and area-based colocalization measurement method was applied. For each mouse, 3-4 sections 120 µm apart were analyzed. Data are presented as fraction of GFAP-positive area overlapping with vimentin-positive area, total GFAP-positive or total vimentin-positive area relative to total ROI area (%).

C1q immunolabeled sections were imaged with an 40x objective (LSM700, Carl Zeiss, NA 1.3) in the peri-infarct motor (M) and somatosensory (S) cortex at 1024 × 1024 pixel resolution. Images from a 160 × 160 µm optical field corresponding to the distance of 20–200 µm (proximal regions: ipsi M and ipsi S1) and 200–400 µm (distal region: ipsi S2) from the infarct border were taken each at superficial (I–IV) and deep (V–VI) cortical layers. Three sections per mouse were analyzed (with Fiji/Image J, v. 2.9.0), whereby the data for the superficial and deep cortical layers were averaged. Data are presented as C1q-positive area relative to the total image area (%).

Clec7a immunolabeled sections were imaged with 20x objective (LSM700, Carl Zeiss, NA 1.3) in the peri-infarct motor (M) and somatosensory (S) cortex at 1024 × 1024 pixel resolution. Field of view location corresponded to the distance of 20–200 µm (proximal regions: ipsi M1 and ipsi S1) and 200–500 µm (distal region: ipsi S2) from the infarct border. Two sections per mouse were analyzed (with Fiji/Image J, v. 2.9.0), and data are presented as Clec7a-positive cell density (Clec7a$^+$ cells/mm^2).

Sections immunolabeled for C3aR, VCAM1, THBS4 or IGF-1 and cell-type specific markers were sequentially scanned with a confocal microscope (LSM 700, Carl Zeiss) using 20x (C3aR and VCAM1) or 40x objective (THBS4 and IGF-1), across 7-15 µm of uniformly labeled tissue volume (8-16 optical sections at 0.93-µm intervals). The resulting z-stack was used to produce maximum intensity projections for each channel. Images were taken in proximal peri-infarct motor or somatosensory cortex within 320 µm × 320 µm or 160 × 160 µm optical field, with 20x and 40x objective respectively. For VCAM1 immunolabeling, images

were also obtained from sections of ipsilesional hippocampus and ventro-basal thalamus (both at Bregma level: -1.8 mm) in stroked mice or ventral horn grey matter of *wobbler* mouse cervical spinal cord.

For assessment of relation between expression of THBS4 and GFAP, Particle analysis plugin (Fiji/Image J, v. 2.9.0) was used. After thresholding of fluorescence signal on maximum intensity projections in THBS4 channel images, the resulting mask of THBS4-positive objects (corresponding to whole cells) was saved as a set of regions of interests (ROIs). Next, the ROIs were superimposed on maximum intensity projections of corresponding GFAP channel images, average GFAP signal intensity within each THBS4-positive ROI was measured and correlated to the area of this ROI. A total of 50 cells (ROIs) from three images were analyzed.

All image acquisitions and quantifications were done by experimenter blinded to experimental group.

RNA isolation

Tissue for gene expression analysis was prepared as described (85) with modifications. Briefly, brains from male mice at P7, P14 (WT and $C3aR^{-/-}$), and P28 (WT) or naive mice (WT) were retrieved and rapidly frozen in isopentane chilled with dry ice and stored at –80°C. Brains were cut into 1 mm-thick slices with a brain matrix and razor blades that were kept cold with dry ice. Peri-infarct regions of primary motor and somatosensory cortex, homotopic regions in the contralesional cortex, or corresponding uninjured cortex in naive mice were dissected from the resulting slices with a 1 mm ID tissue micropuncher (Reusable Rapid Punch kit, WPI) in a set-up that was kept cold with dry ice (85) and stored at –80° C. Tissue micropunches were lysed with Qiazol reagent (Qiagen) and homogenized by passing the lysate 10 times through a microsyringe fitted with a 23G needle and repeating this procedure with a 27G needle. Total RNA was extracted with an RNAeasy Mini Lipid kit (Qiagen), including the on-column

DNAase I digestion step, according to the manufacturer's recommendations. The concentration and purity of isolated RNA were assessed with a Nanodrop 1000 spectrophotometer (Thermo Fisher Scientific).

Library preparation, sequencing, and processing

Samples from WT naive mice and from mice at P7 and P14 were selected for RNA sequencing. RNA integrity was monitored with a Fragment Analyzer system using RNA fragment kit (Agilent Technologies). The RNA quality number (RQN) of all samples was >7. Sequencing libraries were prepared from 100 ng of total RNA with a QuantSeq 3′ mRNA-Seq Library Prep Kit FWD for Illumina (Lexogen) supplied with a UMI Second Strand Synthesis Module for QuantSeq FWD (Lexogen) according to the manufacturer's protocol. Libraries were quantified using Qubit dsDNA assay kit and Qubit 2 fluorometer (Thermo Fisher Scientific) and their quality was assessed with a Fragment Analyzer system using NGS Fragment Kit (Agilent Technologies). Libraries were sequenced on a NextSeq 500 instrument using NextSeq 500/550 High Output Kit v2.5 (75 cycles, Illumina) in 1 x 86bp mode. A median of 5.5 M reads per sample was obtained.

The quality of raw sequencing reads was controlled by FastQC v0.11.9 and screened for potential contamination by FastQ_Screen v0.11.1 (86). 6-bp long UMI tags followed by a "TATA" spacer were extracted from reads with umi_tools v1.0.1 (87) and the command "extract"; the pattern of sequence was defined as "^(?P<umi_1>.{6})(?P<discard_1>TATA){e<=1}". Reads without a spacer were removed. Low-quality reads and adaptors were removed with TrimmomaticSE v0.36 (88), using parameters "ILLUMINACLIP:Lexogen_quantseq.fa:2:30:10 LEADING:3 TRAILING:3 SLIDINGWINDOW:4:15 MINLEN:36". Ribosomal and mitochondrial reads were filtered out with sortmerna v2.1b (89). BAM files with aligned reads were generated with STAR v2.7.3a (90) using the reference *Mus musculus* genome version GRCm38. Reads in the BAM file were

indexed with samtools v1.7 (91, 92) and deduplicated based on the UMI sequence, using umi_tools dedup. Final count tables were generated with Python script htseq-count v0.11.4 (93) using annotation genecode.vM8.gtf and parameter “-m union”.

Differential expression analysis

Differential expression was analyzed with the R package DEseq2 v.1.30.1 (94). Counts were normalized with the command “DESeq”, and the count matrix was limited to data of compared samples. Genes with an average normalized number of reads <20 were removed. Differentially expressed genes (false-discovery rate–adjusted $P < 0.1$ and $\log_2$FC < –0.5 for downregulation and > 0.5 for upregulation) were plotted with EnhancedVolcano package v1.8.0 (95). Functional enrichment analysis for biological processes within gene ontology terms was done with a web-based toolkit, WebGEstAlT 2019 (webgestalt.org; (96) using default parameters and visualized as a bar graph in “weighted set cover” mode. Gene score was calculated for every gene using DESeq2 output as $-\log_{10}(p_{val})$ multiplied by $\log_2$FC and the gene list ranked according to gene score was submitted annotated with entrez IDs. Unadjusted p values were used for gene score calculation due to modest fold-change differences that resulted in a low number of genes with a false-discovery rate–adjusted P value <0.1, precluding meaningful enrichment analysis.

Cellular deconvolution analysis

Cellular deconvolution was done using the CibersortX algorithm at cibersortx.stanford.edu (97). The dataset of Habib et al. (36) served as a reference, encompassing count matrix and metadata labels. To gain perspective on the global map of the major cell populations, the 23 original clusters were merged into 10 classes (11 classes of glutamatergic neurons merged as “Glutamatergic neurons”, 3 GABAergic neuron classes merged as “GABAergic neurons”, and 2 astrocytic populations merged as “Astrocytes”). The reference matrix was built out of 10,000 randomly selected single nuclei and a gene list of 18,438 features from Habib et al. (36). For

each cell population, >300 nuclei were counted. The units of the reference matrix were UMI counts. The scRNA-seq signature matrix was counted in default mode (quantile normalization disabled, minimal expression of 0.75, replicates of 5, sampling of 0.5). Imputations of cell fractions and group-mode expression were used in default settings, with S-mode batch correction enabled, quantile normalization disabled, and n = 100 permutations for significance analysis. The sample mixture file was submitted with an unfiltered gene list (42,809 features) and in UMI counts.

A new signature matrix was generated to obtain an in-depth perspective on subpopulations of astrocytes. The original reference matrix was a subset for astrocytes based on original clustering in Habib et al. (36) and processed with Seurat (v4.0.4). Astrocytic nuclei expressing more than 400 unique genes were normalized with an SCTransform function, yielding a final reference matrix containing 7716 astrocytic nuclei, and the 2000 most variable genes. The original astrocyte clusters (clusters 1–6) were simplified into four groups: clusters 1 and 2 as "$GFAP^{low}$", clusters 3 and 5 as "Intermediate", cluster 6 as "$GFAP^{high}$", and cluster 4 as "DAA". Deconvolution was done with the default settings described above. The reference matrix and the sample matrix were provided in UMI counts.

Overrepresentation analysis for deconvoluted DAAs and $GFAP^{low}$ expression profiles was done in WebGEstAlT (www.webgestal.org) (96), using the most strongly expressed genes [gene expression profile (GEP) score >90] specific for each subpopulation, i.e., genes that were negligibly expressed in other astrocyte subpopulation (GEP score <10). For $GFAP^{low}$ subpopulation, genes with high GEP scores in the Intermediate subpopulation were allowed due to the paucity of highly expressed $GFAP^{low}$-unique genes (Figure S1E, online resource) and the high positive correlation in GEPs between these two subpopulations (Figure S1B, online resource). Gene lists were submitted as gene symbols/entrez IDs, and a built-in murine

genome protein-coding database was chosen as reference. Gene ontologies were considered significantly enriched at a false-discovery rate–adjusted *P* value <0.05.

Quantitative RT-PCR

cDNA from 500 ng of RNA per sample was generated with a GrandScript cDNA synthesis kit (TATAA Biocenter, Gothenburg, Sweden) as recommended by the manufacturer. After cDNA synthesis, samples were diluted to 10 ng/µl of total RNA with nuclease-free water. This concentration was deemed optimal to achieve an assay-specific PCR efficiency of 99–100% for all genes, as judged from dilution series experiments for each of the primer pairs. Formation of a single PCR product for each of the genes was verified by melt curve analysis; the absence of primer dimers was confirmed by the lack of amplification of negative (no template) controls. A total of 1 pmol of forward and reverse primers, respectively (corresponding to working concentration of 0.2 nM in the final reaction volume), was applied to a 384-well PCR plate and air dried overnight. The following primer pairs used: *C3ar1*, FWD 5′-TGTTGGTGGCTCGCAGAT 3′ and REV 5′ GCAATGTCTTGGGGTTGAAA 3′; *Thbs4*, FWD 5′ CGACTTGGTGTGTTCTGCTT 3′ and REV 5′ AGTTGTGGGATTGCTTCTTG 3′; *Igf1*, FW 5′ GACAAACAAGAAAACGAAGC 3′ and REV 5′ ATTTGGTAGGTGTTTCGATG 3′; *Bdnf*, FWD 5′ TCATACTTCGGTTGCATGAAGG 3′ and REV 5′ AGACCTCTCGAACCTGCCC 3′. Single assay primers for *Hprt1, Actb,* and *Arbp* (TATAA Biocenter) served as reference genes. Quantitative RT-PCR reaction mixtures were prepared in a solution of 2.5 µl of TATAA SYBR GrandMaster mix (TATAA Biocenter), 1 µl of diluted template, and 1.5 µl of water and run in duplicate. Real-time PCR experiments were done with a Quant Studio 12K Flex unit (Applied Biosciences, Thermo Fisher Scientific,) using the following temperature profile: 50°C for 2 min and 95°C for 10 min, followed by 40 cycles at 95°C for 15 sec and 60°C for 1 min. To allow normalization of results of different plates, raw CT values were first adjusted with an inter-plate calibrator (TATAA Biocenter) on each

plate, according to the manufacturer's instructions. Of the three reference genes (*Actb, Arbp*, and *Hprt1*), *Hprt1* was identified by NormFinder (https://moma.dk/normfinder-software; (98) as the gene with the most stable expression across all samples. Expression relative to that of *Hprt1* was analyzed with Livak's method (99) and reported as fold change relative to naive WT mice.

Statistical analysis

Data are presented as individual values and mean ± SEM. Comparisons were made between time-points relative to stroke induction, the contralesional and ipsilesional cortex, *C3aR*$^{+/+}$ and *C3aR*$^{-/-}$, WT and *GFAP-C3a*, and PBS- and C3a-treated mice. Gaussian distribution of the data was verified with D'Agostino-Pearson's test. Imaging and quantitative gene expression (by RT-qPCR) data and estimated cell-type fractions from the deconvolution analysis were analyzed by two-way ANOVA followed by planned comparisons, using Sidak's method for three or more groups or paired *t* test for two groups. Round beam test data were analyzed by two-way ANOVA with repeated measures (Figure 1 and 2). Due to single time-point data exclusions, mixed-effects modeling approach (residual maximum likelihood) with time and treatment as fixed factors and subjects as random factor was used for grid walk and cylinder test (Figure 5). Sidak's correction was used to adjust for multiple comparisons. Association between relative GFAP-positive area and behavioral performance was determined by Pearson's coefficient of correlation and linear regression. Due to missing single time-point data, MRI data were analyzed with a mixed-effects model (residual maximum likelihood) with time and treatment as fixed factors and subjects as random factor. False-discovery rate corrections for multiple comparisons using the method of Benjamini and Hochberg. The association between the relative GFAP-positive area and fractional anisotropy, and between the intensity of GFAP immunofluorescence and THBS4-positive area was determined by Pearson's coefficient of correlation.

All analyses were two-tailed, and $P < 0.05$ was considered statistically significant unless stated otherwise. Analyses were done in Prism v. 8.01 (GraphPad) and R v.4.0.5.

Due to anesthesia complications during MRI we excluded the data from 3 and mice treated with C3a and PBS, respectively. We excluded the cylinder test data from mice that made <5 paw touches at a given time-point (P7: 2 and 2 mice, P28: 2 and 3 mice, P56: 2 and 2 mice in the PBS and C3a group, respectively) and the grid walk test data from 1 mouse in the C3a group that showed no initial impairment (0 faults at P7).

76. Kildsgaard J, Hollmann TJ, Matthews KW, Bian K, Murad F, and Wetsel RA. Targeted disruption of the C3a receptor gene demonstrates a novel protective anti-inflammatory role for C3a in endotoxin shock. *J Immunol.* 2000;165:5406-9.

77. Boos L, Campbell IL, Ames RS, Wetsel RA, and Barnum SR. Deletion of the complement anaphylatoxin C3a receptor attenuates, whereas ectopic expression of C3a in the brain exacerbates, experimental autoimmune encephalomyelitis. *J Immunol.* 2004;173:4708-14.

78. Vahidy F, Schäbitz WR, Fisher M, and Aronowski J. Reporting Standards for Preclinical Studies of Stroke Therapy. *Stroke.* 2016;47(10):2435-8.

79. Porritt MJ, Andersson HC, Hou L, Nilsson A, Pekna M, Pekny M, et al. Photothrombosis-induced infarction of the mouse cerebral cortex is not affected by the Nrf2-activator sulforaphane. *PLoS One.* 2012;7(7):e41090.

80. Roome RB, and Vanderluit JL. Paw-dragging: a novel, sensitive analysis of the mouse cylinder test. *JoVE.* 2015(98):e52701.

81. Wang Q, Ding SL, Li Y, Royall J, Feng D, Lesnar P, et al. The Allen Mouse Brain Common Coordinate Framework: A 3D Reference Atlas. *Cell.* 2020;181(4):936-53.e20.

82. Pallast N, Diedenhofen M, Blaschke S, Wieters F, Wiedermann D, Hoehn M, et al. Processing pipeline for atlas-based imaging data analysis of structural and functional mouse brain MRI (AIDAmri). *Front Neuroinform.* 2019;13:42.

83. Yushkevich PA, Piven J, Hazlett HC, Smith RG, Ho S, Gee JC, et al. User-guided 3D active contour segmentation of anatomical structures: significantly improved efficiency and reliability. *Neuroimage.* 2006;31(3):1116-28.

84. Moser JM, Bigini P, and Schmitt-John T. The wobbler mouse, an ALS animal model. *Mol Genetics Genomics.* 2013;288(5-6):207-29.

85. Wager-Miller J, Murphy Green M, Shafique H, and Mackie K. Collection of frozen rodent brain regions for downstream analyses. *JoVE.* 2020(158):doi: 10.3791/60474.

86. Wingett SW, and Andrews S. FastQ Screen: A tool for multi-genome mapping and quality control. *F1000Research.* 2018;7:1338.

87. Smith T, Heger A, and Sudbery I. UMI-tools: modeling sequencing errors in Unique Molecular Identifiers to improve quantification accuracy. *Genome Res.* 2017;27(3):491-9.

88. Bolger AM, Lohse M, and Usadel B. Trimmomatic: a flexible trimmer for Illumina sequence data. *Bioinformatics (Oxford, England).* 2014;30(15):2114-20.

89. Kopylova E, Noé L, and Touzet H. SortMeRNA: fast and accurate filtering of ribosomal RNAs in metatranscriptomic data. *Bioinformatics (Oxford, England).* 2012;28(24):3211-7.

90. Dobin A, Davis CA, Schlesinger F, Drenkow J, Zaleski C, Jha S, et al. STAR: ultrafast universal RNA-seq aligner. *Bioinformatics (Oxford, England).* 2013;29(1):15-21.

91. Li H, Handsaker B, Wysoker A, Fennell T, Ruan J, Homer N, et al. The Sequence Alignment/Map format and SAMtools. *Bioinformatics (Oxford, England).* 2009;25(16):2078-9.

92. Li H. A statistical framework for SNP calling, mutation discovery, association mapping and population genetical parameter estimation from sequencing data. *Bioinformatics (Oxford, England).* 2011;27(21):2987-93.

93. Anders S, McCarthy DJ, Chen Y, Okoniewski M, Smyth GK, Huber W, et al. Count-based differential expression analysis of RNA sequencing data using R and Bioconductor. *Nat Protoc.* 2013;8(9):1765-86.

94. Love MI, Huber W, and Anders S. Moderated estimation of fold change and dispersion for RNA-seq data with DESeq2. *Genome Biol.* 2014;15(12):550.

95. Blighe K, Rana S, and Lewis M. EnhancedVolcano: Publication-ready volcano plots with enhanced colouring and labeling.

96. Liao Y, Wang J, Jaehnig EJ, Shi Z, and Zhang B. WebGestalt 2019: gene set analysis toolkit with revamped UIs and APIs. *Nucl. Acids Res.* 2019;47(W1):W199-w205.

97. Newman AM, Steen CB, Liu CL, Gentles AJ, Chaudhuri AA, Scherer F, et al. Determining cell type abundance and expression from bulk tissues with digital cytometry. *Nat Biotechnol.* 2019;37(7):773-82.

98. Andersen CL, Jensen JL, and Orntoft TF. Normalization of real-time quantitative reverse transcription-PCR data: a model-based variance estimation approach to identify genes suited for normalization, applied to bladder and colon cancer data sets. *Cancer Res.* 2004;64:5245-50.

99. Livak KJ, and Schmittgen TD. Analysis of relative gene expression data using real-time quantitative PCR and the 2(-Delta Delta C(T)) Method. *Methods (San Diego, Calif).* 2001;25(4):402-8.

Supplemental Figures

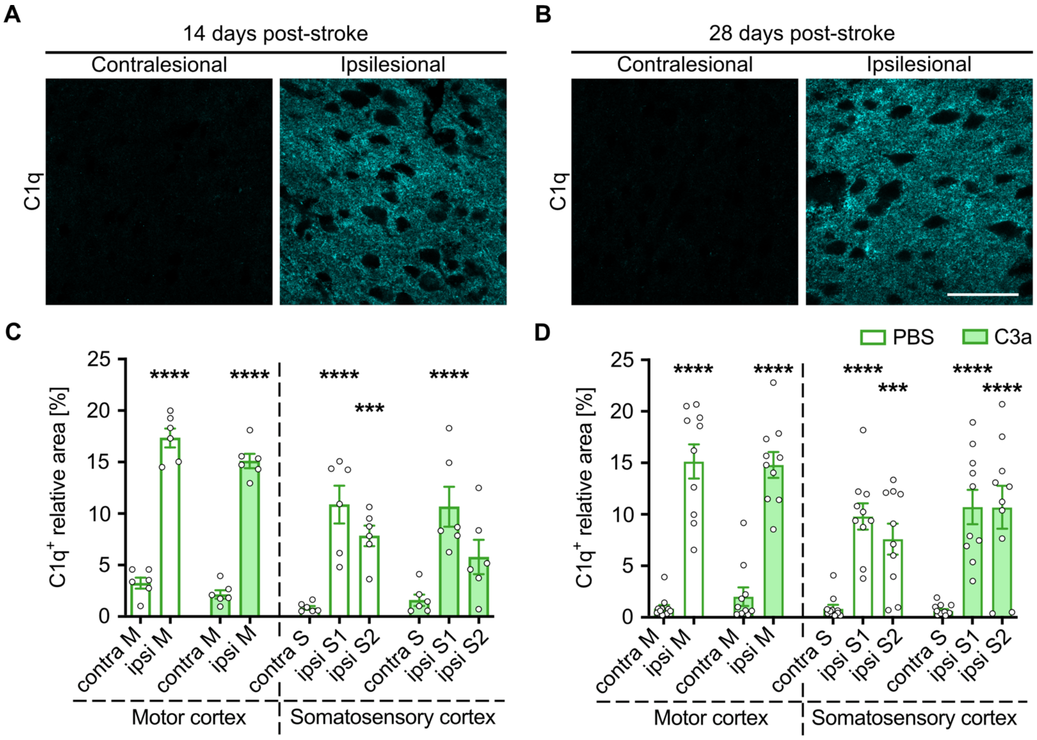

Supplemental Figure 1. Intranasal C3a treatment does not affect the expression of C1q in the post-stroke brain.

Representative images of ipsilesional and contralesional cortex of mice treated with C3a. Tissues were stained with antibodies against C1q on P14 (**A**) and P28 (**B**), cortical regions were chosen for analysis as shown in Figure 3D. Scale bar, 100 µm. Area of C1q positive tissue in the proximal peri-infarct and contralesional cortex of mice treated with PBS or C3a on P14 (**C**) or P28 (**D**). P14: PBS, *n* = 6; C3a, *n* = 6. P28: PBS, *n* = 10; C3a, *n* = 10. Bar plots represent mean ±SEM. Two-way ANOVA with Sidak's planned comparisons: ***$P < 0.001$, ****$P < 0.0001$ for ipsilesional vs. contralesional comparisons. contra, contralesional; ipsi, ipsilesional; M, motor cortex; S, somatosensory cortex;

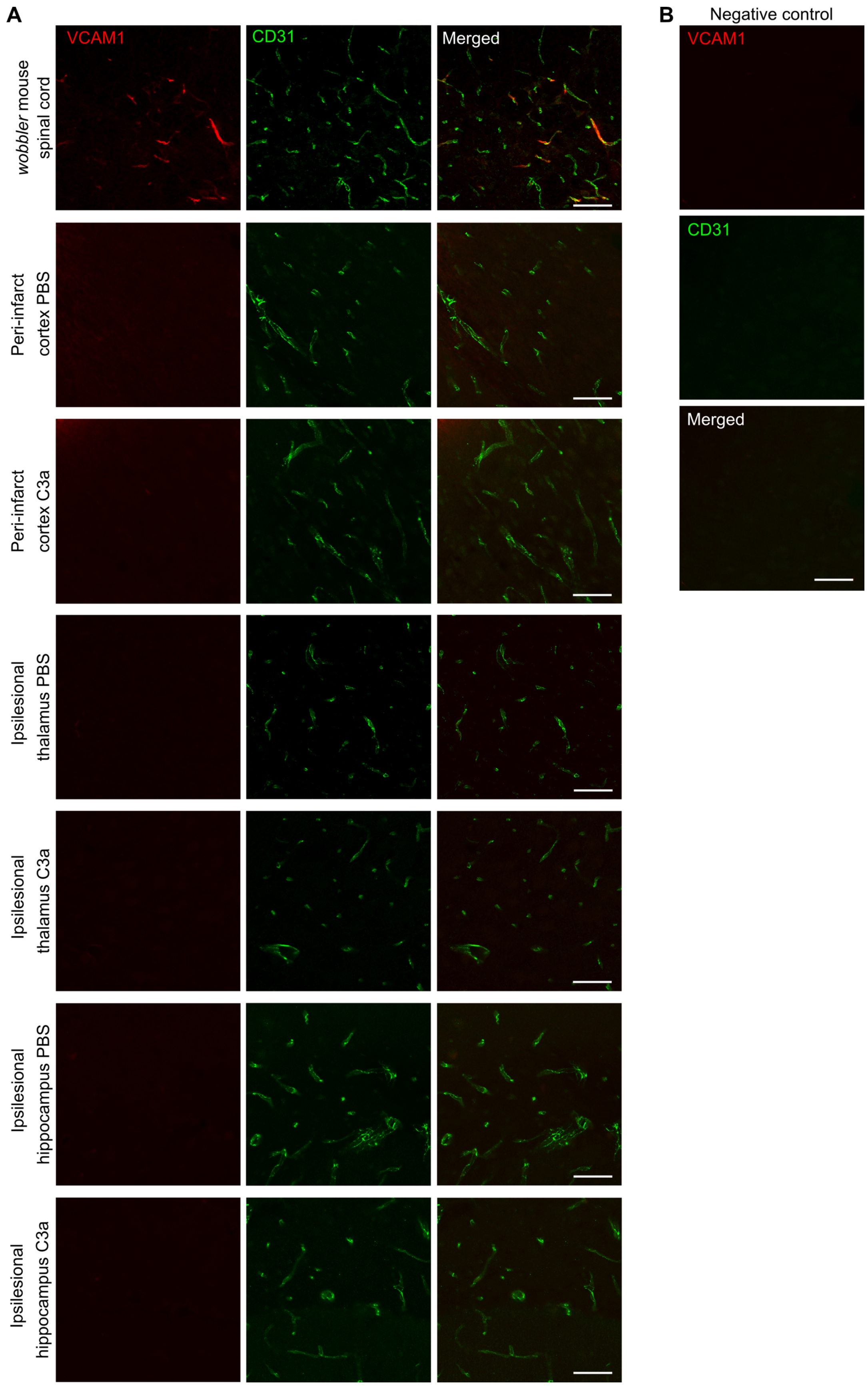

Supplemental Figure 2. Intranasal C3a treatment does not induce the expression of VCAM1 in the post-stroke brain. Representative images of (**A**) wobbler mouse cervical spinal cord (positive control), peri-infarct cortex, ipsilesional thalamus, ipsilesional hippocampus of C3a and PBS treated

mice 28 days after stroke immunostained with antibodies against VCAM1 and CD31. (**B**) Negative control immunostaining without primary antibodies. Scale bar, 50 μm

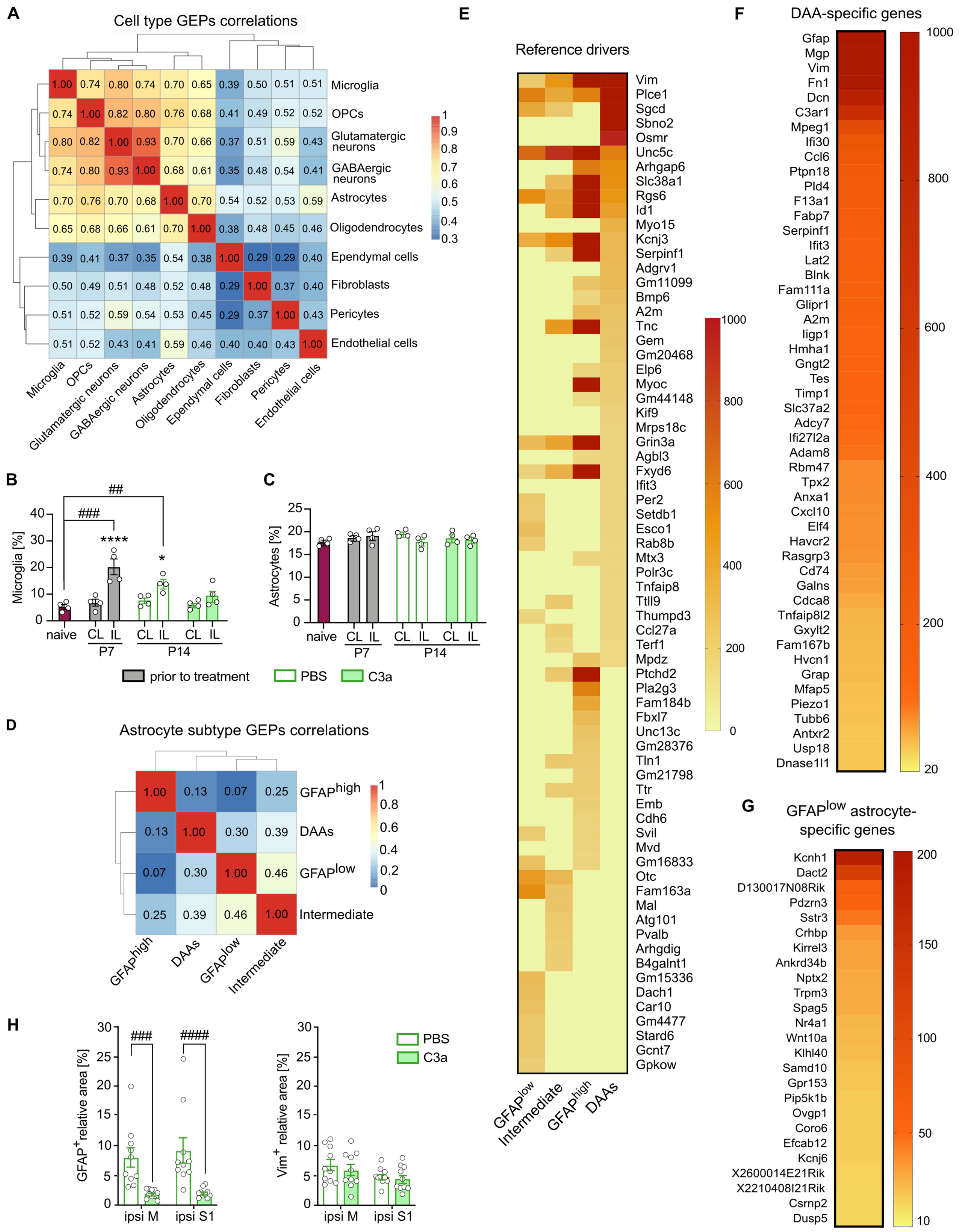

Supplemental Figure 3. Cellular deconvolution of bulk RNAseq data against the single-nucleus RNA dataset generated by Habib et al. (2020). (**A**) Correlation matrix of gene expression profiles (GEPs) for the major brain cell types. (**B**) Sample variance and statistical analysis of the microglia fraction (related to Figure 5D). Bar plots represent mean ± SEM; n = 4 per group and time point. (**C**) Sample variance and statistical analysis of the astrocyte fraction (related to Figure 5D). Bar plots represent mean ± SEM. n = 4 per group and time point. Two-way ANOVA with Sidak's planned comparisons: $^{*}P < 0.05$, $^{***}P < 0.001$ for ipsilesional versus contralesional comparisons; $^{\#\#}P < 0.01$, $^{\#\#\#}P < 0.001$ for comparisons between time points. IL, ipsilesional cortex; CL, contralesional cortex. (**D**)

Correlation matrix of GEPs for astrocyte subpopulations (in the astrocyte-specific deconvolution analysis). (**E**) Reference genes driving the deconvolution analysis of astrocyte subpopulations. Heatmap color scale corresponds to reference score. (**F** and **G**) Genes specifically expressed in DAA (**F**) and *GFAP*^{low} (**G**) astrocytes (i.e., expressed negligibly or not at all in other astrocyte subpopulations). Heatmap color scale corresponds to expression level expressed as counts per million. (**H**) Relative GFAP positive (left) and Vim positive (right) area in the proximal peri-infarct motor (ipsi M) and somatosensory cortex (ipsi S1) of PBS- and C3a- treated mice on P21. Bar plots represent mean ± SEM. PBS, $n = 10$; C3a $n = 10$. Two-way ANOVA with Holm-Sidak's post-hoc test. $^{\#\#\#}P < 0.005$, $^{\#\#\#\#}P < 0.001$.

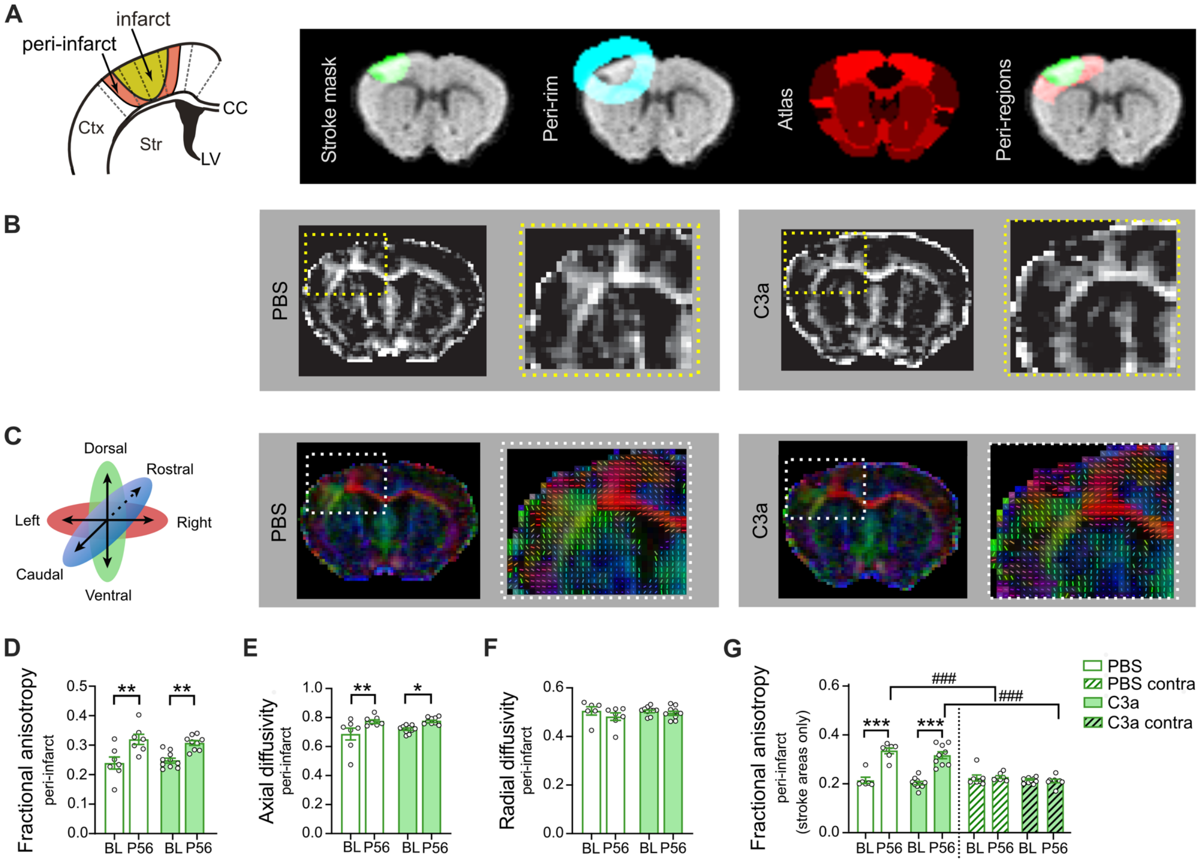

Supplemental Figure 4. Intranasal treatment with C3a does not affect the stroke-induced increase in peri-infarct fractional anisotropy. (**A**) Schematic of cortical regions chosen for DTI analysis and the workflow to generate individual peri-infarct masks. (**B**) Voxelated fractional anisotropy map from representative PBS- and C3a-treated mice. Insets show ipsilesional cortex. (**C**) DTI colour map illustrating the color-coded main diffusion direction per voxel from representative PBS- and C3a-treated mice. Insets show ipsilesional cortex. (**D–G**) Peri-infarct fractional anisotropy (**D**), axial diffusivity (**E**), radial diffusivity (**F**), and fractional anisotropy (**G**) in peri-infarct regions in stroke-affected and corresponding contralesional cortical areas (SSp-ul/ll/un and MOp) before induction of stroke (BL) and on P56. Bar plots represent mean ± SEM. PBS, $n = 7$; C3a $n = 10$. Two-way mixed-effects analysis with false-discovery rate correction: $^{***}P < 0.001$ for comparisons between the time points; $^{\#\#\#}P < 0.001$ for ipsilesional vs. contralesional comparisons.

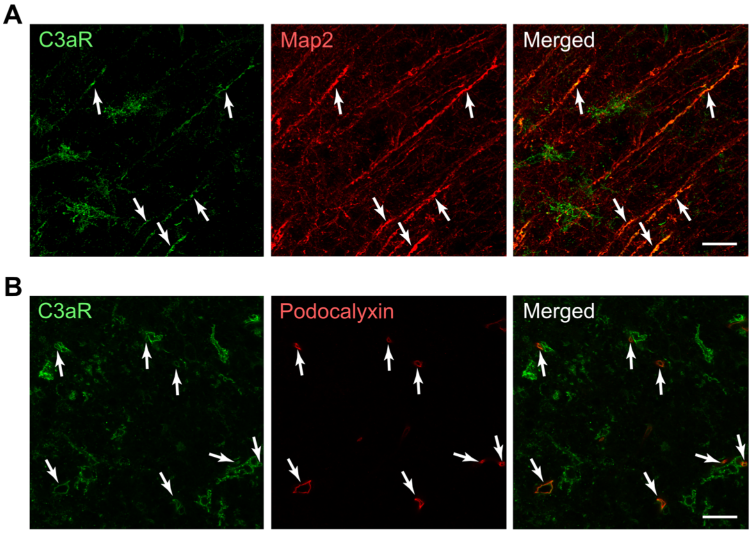

Supplemental Figure 5. C3aR is expressed at low levels by Map2-positive neurons and podocalyxin-positive endothelial cells. Representative images of peri-infarct cortex immunostained with antibodies against C3aR, Map2 and podocalyxin. Arrows point to areas of overlapping immunoreactivity. Scale bar, 20 μm

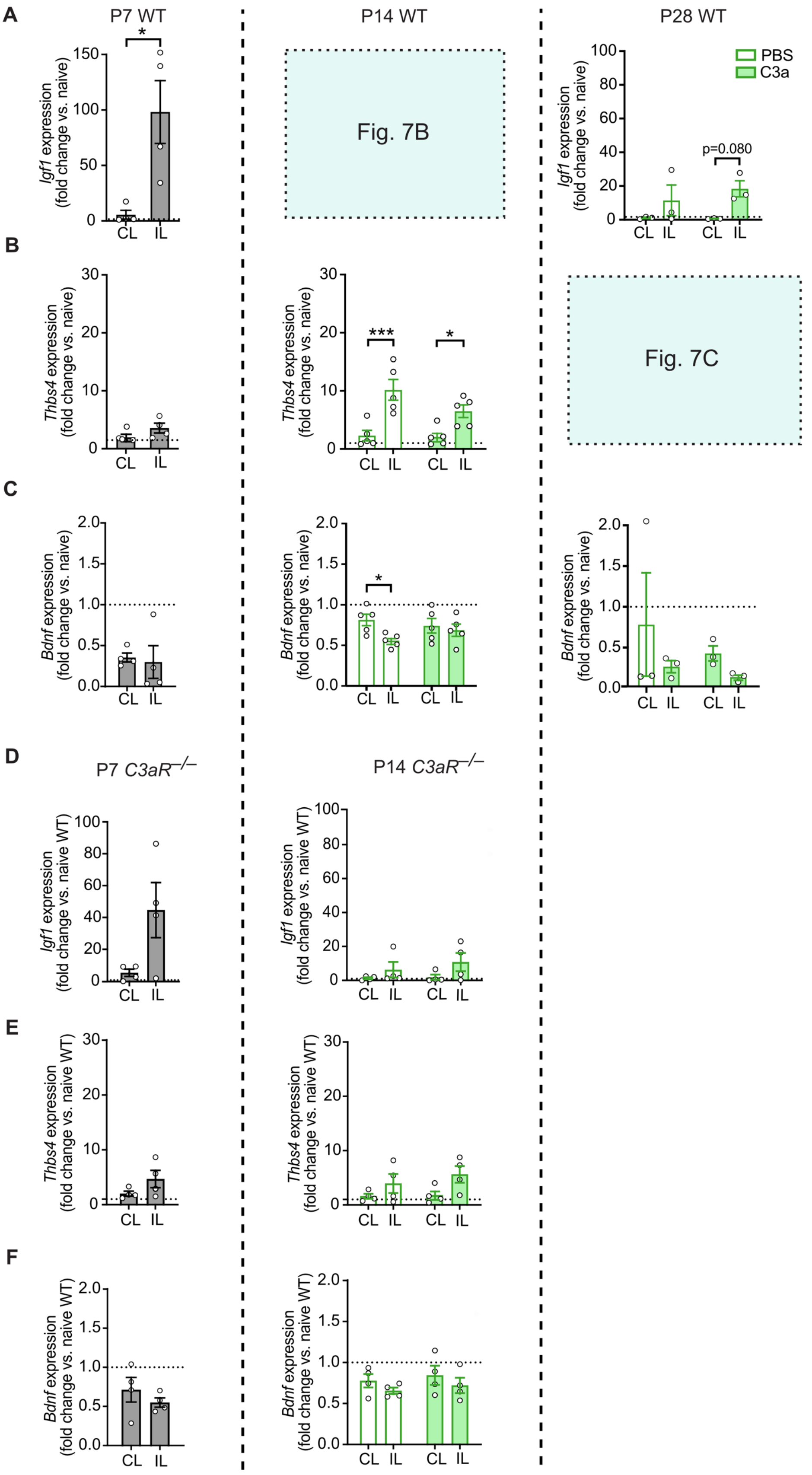

Supplemental Figure 6. Intranasal C3a treatment upregulates peri-infarct expression of neural plasticity–associated genes in WT but not *C3aR*$^{-/-}$ mice. Relative expression of *Igf1* (**A**, **D**), *Thbs4* (**B**, **E**), and *Bdnf* (**C**, **F**) mRNA in peri-infarct (IL) and homotopic contralesional (CL) cortex of WT (**A**–

C) and *C3aR*$^{-/-}$ (**D**–**E**) mice on P7 (before treatment), P14 (after 7 days of intranasal treatment with C3a or PBS), and P28 (end of treatment). Data are expressed as fold change in expression relative to expression in naive WT mice. Bar plots represent mean ± SEM. WT mice: naive, P7, and P28, n = 4 per group; P14, $n = 5$ per group. *C3aR*$^{-/-}$ mice: P7 and P14, $n = 4$ per group. Paired t test (P7 analysis) or two-way ANOVA with Sidak's planned comparisons (P14 and P28 analyses): $^{*}P < 0.05$, $^{***}P < 0.001$ for ipsilesional (IL) vs. contralesional (CL) comparisons.

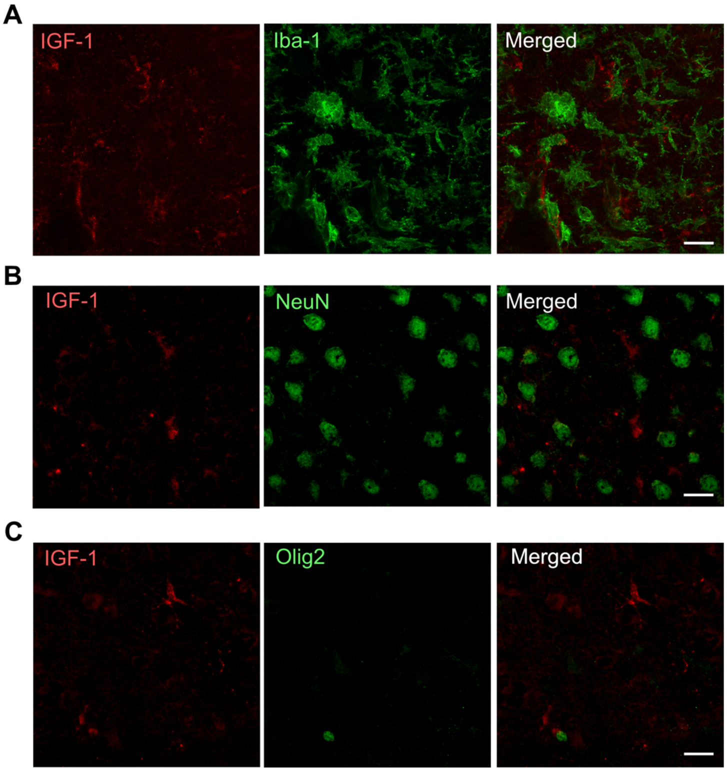

Supplemental Figure 7. In the peri-infarct cortex, IGF-1 is not expressed in Iba-1-positive microglia, NeuN-positive neurons or Olig2-positive oligodendrocytes. Representative images of peri-infarct cortex immunostained with antibodies against IGF-1, Iba-1, NeuN and Olig2. Scale bar, 20 μm.

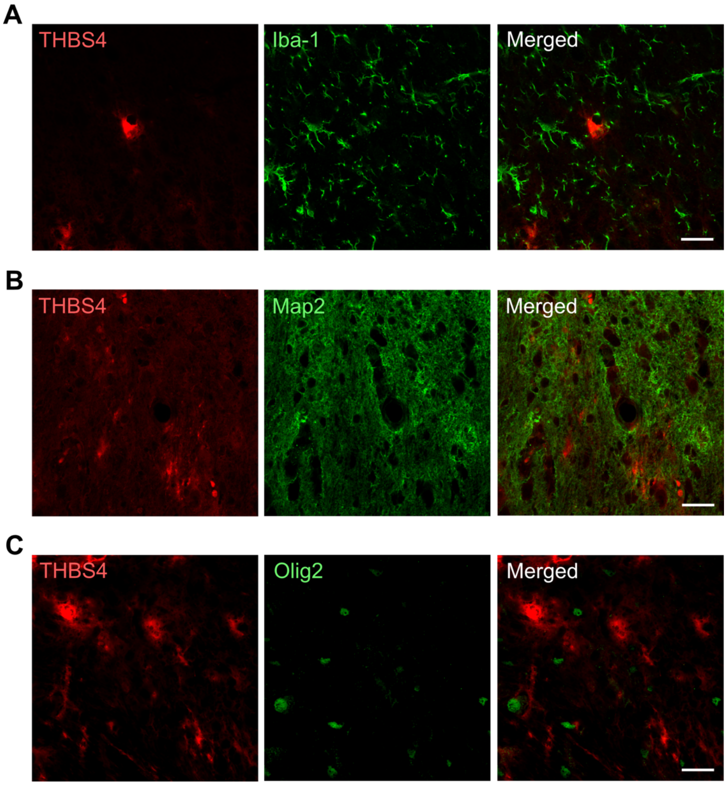

Supplemental Figure 8. Thrombospondin 4 (THSB4) is not expressed in Iba-1-positive microglia, Map2-positive neurons or Olig2-positive oligodendrocytes. Representative images of peri-infarct cortex immunostained with antibodies against THBS4, Iba-1, Map2 and Olig2. Scale bar, 20 μm.
